# Carbon Nanocones Geometry-Dependent Descriptors Evidence Levels and Polymer Interfaces

**DOI:** 10.3390/polym18212644

**Published:** 2026-10-25

**Authors:** Can Doğan Vurdu, Fatma Kandemirli

**Affiliations:** Department of Biomedical Engineering, Faculty of Engineering and Architecture, Kastamonu University, Kastamonu 37150, Türkiye; fkandemirli@kastamonu.edu.tr

**Keywords:** carbon nanocones, apex topology, evidence classification, polymer interfaces, nanocomposites, adsorption, nanohorns, validation

## Abstract

Carbon nanocones (CNCs) couple apex topology, geometry, and edge chemistry to electronic structure and adsorption. This critical narrative review defines a descriptor register that records structural identity, property definition, computational or experimental conditions, source location, and evidence class. Comparisons are restricted by model and method compatibility rather than combined into a universal performance score. Direct experimental evidence is architecture-specific: CNC probes characterize polymers, cone–particle assemblies conduct within adhesives, hollow nanocone arrays perform capacitive deionization, and conical carbon films emit electrons. Indirect experimental evidence from nanohorn–polymer composites addresses dispersion, reinforcement, percolation, and responsive interfaces, but does not validate isolated angle-controlled CNCs. Computational predictions include CNC–polyethylene interphases, chitosan-associated adsorption, electronic tuning, ion storage, and thermal rectification. Experimental polymer-related evidence remains sparse and processing-dependent. Source-resolved comparisons replace untraceable pooled gap, work-function, and adsorption-energy ranges; finite-size and edge-state effects prevent a universal angle-to-property ordering. A validation roadmap connects model convergence and structural characterization to matched controls and application-specific tests. The resulting framework supports traceable interpretation while separating computational promise from measured material and device performance.

## 1. Introduction

Carbon materials form stable structures across multiple dimensionalities, including zero-dimensional fullerenes, one-dimensional carbon nanotubes (CNTs), two-dimensional graphene, and three-dimensional disordered or porous carbon frameworks [1,2,3,4,5,6,7,8,9]. This structural diversity originates from the flexibility of carbon bonding and the stability of sp, sp^2^, and sp^3^ hybridization states across carbon allotropes [1,2,3,4,5,6,7,8,10]. Among sp^2^-dominated nanocarbons, graphene and CNTs have become benchmark materials; however, carbon nanocones (CNCs) occupy a distinct structural position because they combine graphitic bonding with a topologically defined apex and spatially non-uniform curvature field [11,12,13,14].

An ideal continuum CNC is constructed by removing a 60° × n sector from a graphene sheet and joining its edges, with n = 1–5. The corresponding pentagonal disclinations concentrate positive Gaussian curvature at the apex; the smooth sidewall is developable and has zero Gaussian curvature away from the apex, although its extrinsic curvature varies along the cone [11,12,13,14,15,16]. The five full apex angles are 112.9°, 83.6°, 60.0°, 38.9°, and 19.2° for n = 1–5, respectively. The geometric relation is given explicitly in Section 2.1. These angles define ideal topological classes, not unique atom counts, terminations, or electronic states.

The scientific interest in CNCs arises from the coupling between geometry and physical properties. The cone angle controls the strain distribution, apex topology influences electronic localization, edge termination affects frontier electronic states, and surface functionalization modifies adsorption selectivity, charge transfer, and electrochemical activity [14,16,17,18,19,20,21,22,23,24]. These features have motivated studies on CNCs and cone-rich carbon architectures for alkali–ion energy storage [21,22,25,26], gas sensing and molecular detection [23,24,27,28], field emission [17,29,30,31,32], catalysis [20,33,34,35,36,37,38], and optoelectronic, or nonlinear optical responses [28,39,40,41,42,43,44,45,46,47,48,49].

The literature contains ideal finite CNCs, periodic cone-containing models, molecular carboncones, multilayer cone particles, and nanohorn aggregates. Their descriptors cannot be interchanged without specifying the architecture and method [13,14,18,50,51,52,53,54,55,56,57,58,59,60,61,62,63]. This critical narrative review therefore uses an explicit descriptor register and evidence-classification procedure, defined in Section 5.2, Section 5.3 and Section 5.4. It does not claim a systematic meta-analysis, a complete bibliometric census or a universal numerical ranking of nanocarbons.

Polymer relevance extends across distinct evidence levels. Experiments demonstrate CNC-based AFM characterization [61] and conducting cone–particle assemblies within an adhesive [64]. CNC–polyethylene interphase mechanics [65] and chitosan-associated drug adsorption [66] are computational studies. Nanohorn-filled polymers provide a broader experimental basis for local mechanical response, thermal insulation, conducting networks, and responsive gels [67,68,69,70,71,72]. These results are reviewed in Section 9.5 with their architecture, processing, and measurement limits; they do not establish generic reinforcement or heat-conduction gains for isolated, angle-selected CNCs.

## 2. Structural Origin and Classification of Carbon Nanocones

### 2.1. Topological Construction

Removing a sector of angular width nπ/3 leaves a circumferential fraction 1 − n/6. Equating this remaining arc at slant distance l to the cone circumference gives 2πl(1 − n/6) = 2πl sin(α/2). Thus, the full apex angle is [11,12,13,14,15,16]

(1) $$\alpha =2\;arcsin\left(1-\frac{n}{6}\right),\;n=1,\ldots ,\;5$$

Here, α is the full apex angle and α/2 is the angle between the axis and a generator. In particular, n = 2 gives α = 2 arcsin(2/3) = 83.6206…°, rounded to 83.6°. The relation is a continuum construction and does not by itself specify the atomistic tip or edge.

This topological construction links CNCs to other curved carbon allotropes. Fullerenes require 12 pentagons to close a spherical cage, nanotube caps require pentagons to terminate cylindrical walls, and graphitic cones use a localized subset of non-hexagonal defects to generate conical tips [3,7,11,12,13,14,15,16,73]. Thus, CNCs belong to a broader family of curved sp^2^-carbon structures, whose properties are controlled by non-hexagonal defects, curvature localization, aggregation state, and apex topology [11,12,13,14,15,16,54,55,56,57].

**Figure 1:**
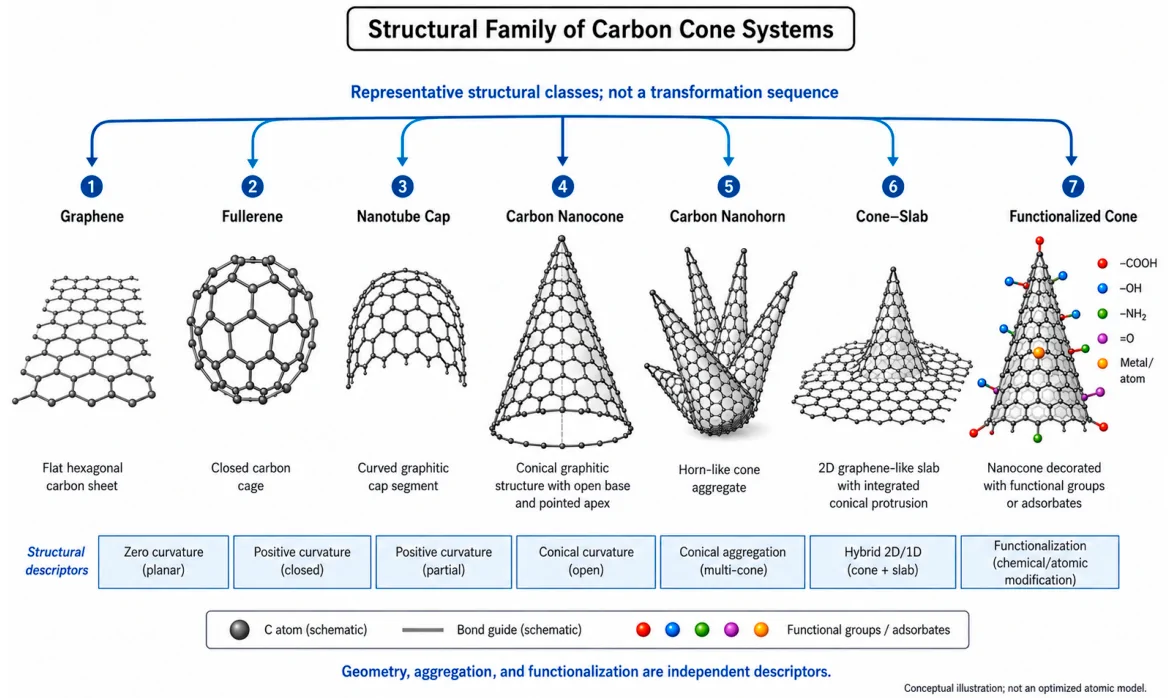
Structural family of carbon-cone systems. The original seven-class composition is retained with revised annotations and an atom/bond legend. Graphene, a closed cage, a tube cap, an isolated cone, a nanohorn aggregate, a cone–slab motif, and a functionalized cone represent different architectures, not a transformation sequence or monotonic curvature ranking. AI-assisted conceptual illustration; not to scale and not an atomistically optimized model [11,12,13,14,15,16,54,55,56,57,63].

Figure 1 retains the seven structural classes of the original illustration. Geometry, aggregation, hybridization, and functionalization are separate structural variables, not steps along a universal curvature or performance sequence. The ball-and-stick motifs identify conceptual architectures rather than optimized atomic coordinates [11,12,13,14,15,16,54,55,56,57,63].

### 2.2. The Five Discrete Cone Angles

Table 1 reports the five ideal geometric classes calculated from Equation (1). A cone angle alone does not specify the number of atoms, relative arrangement of pentagons, open-edge structure, termination, or charge. Those variables must accompany the angle in any electronic or adsorption comparison [14,16,18,50,51,52].

**Figure 2:**
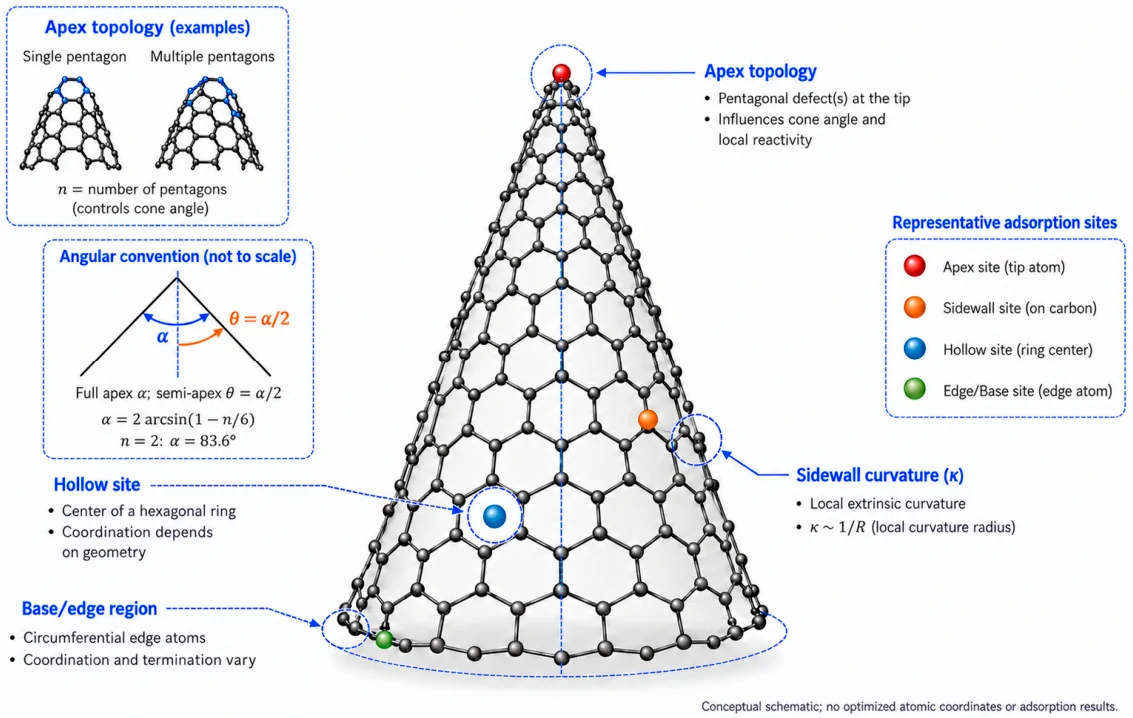
CNC geometry and representative site terminology. The original cone illustration is retained with corrected angle annotations: α is the full apex angle, θ = α/2 is the axis–generator angle, and n = 2 gives α = 83.6° from Equation (1). Hollow denotes a ring-center site; colored markers identify site categories, not computed adsorbate positions. The sidewall curvature label concerns extrinsic curvature, not a nonzero Gaussian curvature of the ideal developable sidewall. AI-assisted conceptual illustration; atomic positions, and bonds are illustrative and not an optimized structure [11,12,13,14,15,16,19].

Figure 2 retains the original cone and site-color scheme while clarifying the full apex angle α and semi-angle θ = α/2. The geometric construction in Equation (1), rather than an apparent ring count in the illustration, determines the five ideal angles. Atomic connectivity, tip reconstruction, and adsorption geometry require a specified coordinate model or microscopy evidence [13,14,15,16,17,18,19].

### 2.3. Apex Topology

The apex is the defining structural element of a CNC. It contains a non-hexagonal defect pattern that localizes the curvature and controls the electronic perturbation near the cone tip. Larger angular deficits produce sharper cones, stronger apex strain, and greater σ–π orbital rehybridization [14,16,18,19]. These features promote localized electronic states near the Fermi level and can enhance the adsorption of metal atoms, ions, and small molecules at the apex or other highly curved sites [17,18,20,21,22].

### 2.4. Edge Configuration

Finite CNCs are strongly affected by their edge configuration [13,18,50,51,52,74,75]. Open-base structures may include zigzag, armchair, mixed, reconstructed, or hydrogen-passivated edges. Unpassivated edges can introduce dangling bonds, spin-polarized states, and artificially high reactivity in the calculations [18,50,51,52,74,75]. Hydrogen passivation is commonly used to isolate the apex and curvature effects, whereas experimentally observed cone-rich carbons may contain reactive edges, structural disorder, surface heterogeneity, and possible oxygen-containing groups, depending on the synthesis and post-treatment conditions [53,58,59,60,61,62,63].

Therefore, edge passivation choices have a major influence on the computed properties of CNCs [18,50,51,52,74,75]. Comparisons between H-terminated and bare-edge models should be made cautiously because dangling-bond artifacts can dominate the electronic structure of small CNC clusters. For this reason, future computational studies should explicitly report the edge structure, passivation scheme, atom count, and whether the observed electronic states originate from the apex, sidewall, or edge region.

## 3. Carbon Nanocones Versus Carbon Nanohorns: Terminology and Experimental Relevance

A clear distinction between carbon nanocones (CNCs) and single-walled carbon nanohorns (SWCNHs) is essential for comparing computational predictions with experimental observations. In the literature, the terms “nanocone” and “nanohorn” are sometimes used in overlapping ways, but they do not necessarily describe the same structure [54,55]. CNCs are most often treated as idealized or atomistically defined conical graphene-derived structures with discrete apex topologies [11,12,13,14,15,16], whereas SWCNHs are experimentally accessible horn-like sp^2^-carbon structures that typically occur as aggregates [54,55,56,57].

This distinction is especially important in application-oriented discussions. Computational CNC models can isolate the effects of the cone angle, apex topology, edge termination, and adsorption site; however, they do not capture the aggregation, porosity, possible oxygen-containing surface groups, interparticle contacts, and sample heterogeneity that characterize experimentally produced SWCNH materials [54,55,56,57]. Therefore, SWCNH experiments provide valuable indirect evidence for cone-rich carbon architectures, but they should not be interpreted as direct validation of isolated, angle-controlled CNC models without additional structural justification [53,54,55,56,57,58,59,60,61,62,63].

### 3.1. Definitions

A carbon nanocone is an idealized or experimentally observed conical graphene-derived structure with a specific apex topology. In ideal topological models, CNCs are characterized by the number of pentagonal defects, n = 1–5, and a corresponding discrete cone angle. Many CNC studies use finite H-passivated clusters or periodic cone-slab models to isolate the effects of the cone angle, apex topology, edge termination, local curvature, and adsorption site [14,16,17,18,19].

A single-walled carbon nanohorn is a horn-like, tubular, or conical sp^2^-carbon structure that typically forms spherical aggregates containing many individual nanohorns. SWCNHs are experimentally accessible and have been produced by CO_2_ laser ablation, arc discharge, and related high-temperature carbon-vaporization methods [54,55,56,57]. Three aggregate morphologies—dahlia-type, bud-type, and seed-type—are commonly discussed in the SWCNH literature and may exhibit different surface areas, porosities, oxidation states, and electrochemical behaviors [54,55,56,57].

The key distinction is that an isolated CNC model represents a controlled atomistic geometry, whereas an SWCNH sample is usually an aggregate material whose measured properties are influenced by horn packing, interparticle contacts, porosity, possible oxygen-containing groups, and defect heterogeneity. Consequently, SWCNH experiments should be considered indirect experimental analogs for cone-rich carbon architectures, rather than direct experimental equivalents of isolated CNC models [53,54,55,56,57,58,59,60,61,62,63].

### 3.2. Key Structural Differences

The distinction between idealized CNC models and experimentally accessible SWCNH aggregates is summarized in Table 2. CNCs are typically treated as atomistically defined conical graphene-derived structures with discrete cone angles and well-defined apex topologies, whereas SWCNHs are experimentally accessible horn-like carbon structures that usually occur as aggregates [11,12,13,14,15,16,17,18,19,54,55,56,57]. This distinction is important because application data obtained from SWCNH aggregates cannot be directly assigned to isolated CNCs without considering aggregation, porosity, surface oxidation, interparticle contacts, and structural heterogeneity [54,55,56,57].

Therefore, SWCNHs provide useful indirect experimental support for cone-rich sp^2^-carbon architectures, but they should not be treated as direct experimental equivalents of isolated CNC models. This distinction is essential when comparing computational descriptors with experimental data. This terminology issue is also reflected in the broader literature, where the terms nanocone and nanohorn are sometimes used interchangeably, despite their important structural differences [54,55]. A clearer distinction improves the comparison between theory and experiment: CNC models are most useful for identifying geometry-controlled electronic and adsorption trends, whereas SWCNH systems are more informative for assessing experimentally accessible aggregate behavior, scalable synthesis, oxidation chemistry, functionalization, and device-level applications [14,16,17,18,19,54,55,56,57].

### 3.3. Bridging Computation and Experiment

Most computational CNC studies model isolated nanocones that do not directly correspond to experimentally available aggregates. These idealized models are useful for isolating the effects of the cone angle, apex topology, edge passivation, adsorption site, local curvature, and electronic structure [14,16,17,18,19,20,21,22,50,51,52]. However, they do not capture aggregation, porosity, possible oxygen-containing surface groups, interparticle contacts, or sample heterogeneity, all of which can strongly influence the measured properties of experimentally accessible cone-rich carbon materials and SWCNH aggregates [54,55,56,57].

In contrast, the SWCNH literature provides one of the closest experimental analogs for cone-rich sp^2^-carbon architectures because SWCNHs are experimentally accessible and have been studied in the context of adsorption, energy storage, biomedical applications, functionalization, and related uses [25,55,56,57,76]. Nevertheless, SWCNHs should be treated as indirect experimental analogues rather than direct validation of isolated CNC models. A DFT model of an H-passivated nanocone represents a controlled atomistic motif, whereas an SWCNH sample represents an aggregate material whose properties are affected by horn packing, accessible surface area, pore structure, surface oxidation, interparticle contacts, and defect distribution [14,16,17,18,19,20,21,22,50,51,52,54,55,56,57].

Therefore, future CNC studies should explicitly state whether their models are intended to represent isolated nanocones, nanohorn tips, cone-shaped defects in larger graphitic architectures, cone-rich films, or porous carbon electrodes. This distinction is essential for comparing computational descriptors with experimental measurements and avoiding overinterpretation of CNC application claims [14,16,17,18,19,20,21,22,50,51,52,54,55,56,57].

## 4. Synthesis and Experimental Characterization

Carbon nanocones and cone-like carbon structures have been produced using arc discharge, laser ablation, chemical vapor deposition, plasma-assisted methods, pyrolysis, and bottom-up molecular approaches [11,12,13,53,56,57,58,59,60,63]. In many experimentally produced samples, conical structures coexist with amorphous carbon, nanohorns, graphitic particles, nanotubes, nanodiscs, and other defective carbon structures. This coexistence makes purification, structural assignment, and quantitative comparison with idealized CNC models experimentally challenging [53,58,59,60]. Atomically precise molecular carboncones have also been synthesized using bottom-up routes; however, such systems should be distinguished from scalable macroscopic CNC powders, films, and electrodes [63].

Transmission electron microscopy (TEM) and high-resolution transmission electron microscopy (HRTEM) provide the most direct evidence of cone morphology, apex structure, graphitic wall arrangement, and cone angle [11,12,13,53,58,59,60,63]. Raman spectroscopy is commonly used to evaluate the graphitic quality and defect density through the D/G-band response, whereas X-ray photoelectron spectroscopy (XPS) can provide information on surface bonding and oxygen-containing groups when surface chemistry is relevant. BET surface area analysis, scanning electron microscopy (SEM), atomic force microscopy (AFM), and electrochemical measurements provide complementary information on the porous structure, surface morphology, mechanical behavior, energy-storage performance, sensing behavior, and electrode-level applications [53,55,57,61,62].

### 4.1. Experimental Milestones

The representative experimental milestones in the development of carbon nanocones, carbon nanohorns, and related cone-rich carbon systems are summarized in Table 3. Early experimental studies established that conical carbon structures can form in graphitic carbon materials. Ge and Sattler reported fullerene-like carbon cones, whereas Krishnan et al. later identified graphitic cones with cone angles consistent with topological predictions [11,12]. Single-walled carbon nanohorns (SWCNHs) were subsequently reported as aggregate materials, providing an experimentally accessible but structurally heterogeneous class of cone-rich carbon architectures [56].

A major recent advance is the rational synthesis of an atomically precise carboncone under mild conditions. Zhu et al. prepared a structurally well-defined carboncone molecular scaffold, C_70_H_20_, and confirmed its molecular architecture by X-ray crystallography [63]. This result is significant because it provides direct experimental access to a single-layer carboncone with a defined atomic structure, partially addressing one of the longstanding limitations in the field [63]. However, this achievement should be interpreted carefully: it represents a molecularly precise carboncone rather than a scalable route to monodisperse macroscopic CNC materials, films, or electrodes. Thus, it is best viewed as a bridge between bottom-up molecular synthesis and the broader, materials-oriented CNC literature [63].

The studies listed in Table 3 demonstrate that conical carbon structures are experimentally accessible in several forms, including graphitic cones, SWCNH aggregates, multiwalled carbon nanocones, atomically precise molecular carboncones, AFM probe structures, and hollow carbon nanocone arrays. However, they also highlight a central limitation of the field: most experimentally available samples are heterogeneous, aggregated, multi-walled, molecular-scale, or application-specific architectures rather than isolated, monodisperse single-walled CNCs with predetermined cone angles, edge terminations, and reproducible apex topologies. This distinction is important because many predicted CNC properties, including work-function reduction, apex-localized electronic states, site-selective adsorption, and geometry-dependent charge transfer, are sensitive to the exact cone geometry [14,16,17,18].

Therefore, although experimental progress has confirmed the feasibility of cone-rich carbon architectures, the controlled synthesis of isolated CNCs with well-defined cone angles and apex topologies remains unresolved at the material scale. Atomically precise molecular carboncones and hollow carbon nanocone arrays represent important advances, but they should not be treated as direct validation of all idealized atomistic CNC models without considering the differences in size, edge structure, aggregation state, substrate interaction, and device architecture.

### 4.2. Persistent Experimental Limitations

The central experimental obstacle in carbon-nanocone research is sample heterogeneity. Computational models specify the exact cone angles, atom counts, edge terminations, and apex defect patterns, whereas experimentally produced samples typically contain distributions of cone sizes, wall structures, residual catalysts, amorphous regions, nanohorns, nanodiscs, graphitic fragments, and possible oxygen-containing surface groups, depending on the synthesis or post-treatment conditions [13,53,58,59,60]. This structural diversity makes it difficult to establish quantitative structure–property relationships between ideal CNC models and the experimentally measured properties of cone-rich carbon samples.

The literature reviewed here indicates that the routine synthesis of isolated single-walled CNCs with a predetermined cone angle, defined apex topology, and controlled edge termination has not yet been demonstrated at the material scale [11,12,13,53,58,59,63]. Although the five-angle quantization predicted by topology has been supported by microscopy, the selective synthesis of a single cone angle remains unresolved in experimentally produced heterogeneous samples [11,12,13,53,58,59]. This limitation is critical because many predicted CNC properties, including apex-localized electronic states, work-function reduction, field enhancement, and site-selective adsorption, depend sensitively on the exact cone geometry [14,16,17,18,19,20].

Accordingly, the statement that the controlled synthesis of isolated carbon nanocones remains unresolved should be interpreted with scale and structural precision in mind. At the molecular scale, atomically precise single-layer carboncones have been synthesized; however, routine material-scale synthesis of isolated, monodisperse CNCs with predetermined cone angles, apex topologies, wall heights, edge terminations, and surface chemistries is still lacking [63]. Most experimentally available cone-rich materials remain heterogeneous and may contain nanohorns, graphitic particles, nested cones, amorphous carbon, nanodiscs, or partially oxidized domains, depending on the synthesis and post-treatment conditions [53,54,55,56,57,58,59,60]. This gap between the theoretical structural precision and experimental heterogeneity remains a central barrier to establishing robust structure–property relationships for CNCs [14,16,17,18,19,20,53,54,55,56,57,58,59,60,61,62,63].

Therefore, CNC application claims should be interpreted in light of the evidence level. Direct experimental results on heterogeneous cone-rich carbons, nanodiscs, hollow nanocone arrays, or SWCNH aggregates provide valuable support for the broader relevance of conical carbon architectures; however, they do not constitute direct validation of idealized isolated CNC models [53,54,55,56,57,58,59,60,61,62,63]. Future experimental progress will require synthesis routes that control cone angle, apex topology, wall structure, edge termination, and surface chemistry within well-characterized samples.

## 5. Computational Modeling of Carbon-Cone Systems

### 5.1. Modeling Approaches

Computational modeling enables the controlled variation of the cone angle, apex topology, edge termination, charge state, dopant configuration, and adsorption site in carbon-cone systems [14,16,17,18,19]. Two main modeling strategies are commonly used: finite-cluster and periodic-slab models.

Finite-cluster models are usually constructed by cutting a wedge from graphene, reconnecting the lattice, passivating the open edges with hydrogen, and relaxing the structure using density functional theory (DFT) or related electronic-structure methods [14,16,17,18,19,22,50,51,52]. These models are convenient for studying localized adsorption, frontier orbitals, molecular descriptors, and apex-specific electronic states. However, small-cluster models may exaggerate edge and quantum-confinement effects, especially when the electronic states of interest are not clearly separated from edge-localized states.

Periodic-slab models embed a cone or cone-like protrusion into a supercell with sufficient vacuum spacing. They are more appropriate for electrode-like systems, band-structure calculations, work-function analysis, and cone-slab geometries. However, periodic calculations require careful convergence with respect to the vacuum thickness, dipole correction, k-point sampling, energy cutoff, and supercell size to avoid artificial image interactions [18,20,77,78,79,80,81,82,83,84,85,86,87].

**Figure 3:**
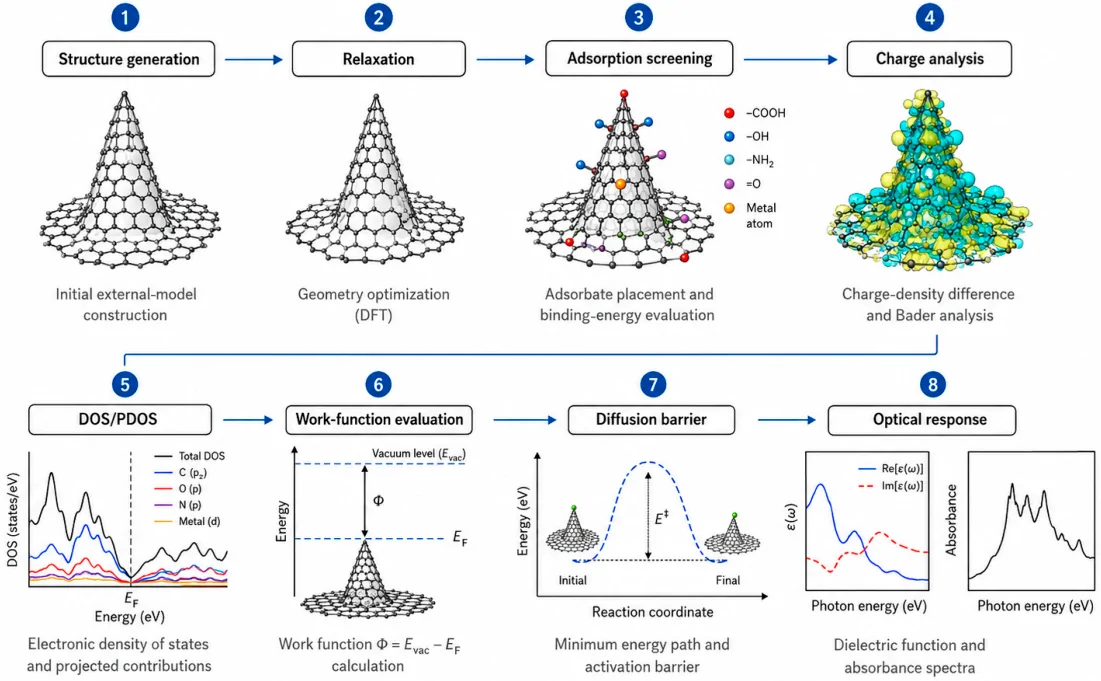
Original AI-generated conceptual illustration of a CNC computational workflow, retained without changing the image. The depicted atomic networks, colored charge isosurfaces, DOS/PDOS curves, barrier profile, and optical curves are explanatory schematics, not calculated or measured results presented by this review. Arrows indicate a possible workflow, not a mandatory calculation sequence. The work-function diagram applies only where the reference and boundary conditions support that definition. E^‡^ denotes the schematic activation barrier. Yellow and cyan distinguish schematic charge-redistribution regions without a calibrated sign or magnitude. DOS/PDOS colors identify the labeled total or atomic-orbital contributions. Blue and red optical curves denote the real and imaginary dielectric components, respectively.

Figure 3 retains the original illustration of a computational workflow: model construction, relaxation, adsorption screening, charge analysis, DOS/PDOS, work-function evaluation, diffusion pathways, and optical response. These operations can generate descriptors for defined models. The evidence register in Table 4 additionally records structural identity, method, source locator, and claim-specific evidence class [14,18,20,22,50,51,52].

### 5.2. Descriptors

The descriptor framework is an operational record, not a weighted score. Each record contains the material architecture and structural variables; the observable, units, and mathematical definition; the computational or experimental protocol; the exact source and locator; and an evidence class relative to the claim being made. Table 4 specifies the minimum fields. Multiple observables from one paper are separate records when their conditions or reference states differ. This register makes an interpretation traceable without treating a favorable adsorption energy as a proxy for a working device.

The adsorption energy is commonly defined as

(2) $$E_{ads}=E_{cone+adsorbate}-E_{cone}-E_{adsorbate},$$

where the terms refer to the combined system, the host, and the explicitly specified adsorbate reference, respectively. A negative electronic binding energy indicates stabilization relative to that reference; it is not itself a finite-temperature adsorption-free energy or proof of stability against bulk-metal aggregation. Charge state, stoichiometry, and electronic-reference conventions must be conserved. Atomic-H binding, molecular-H_2_ adsorption and total multi-H hydrogenation energies are different quantities. Dispersion treatment matters particularly for weak molecular physisorption [88,89,90,91,92,93].

For charge-transfer analysis, Bader charge partitioning and charge-density-difference maps are frequently used to quantify electron redistribution between the adsorbate and the cone framework [94,95,96]. For work function calculations, the work function is usually estimated from the difference between the vacuum and Fermi levels, and slab or cluster calculations require careful treatment of the vacuum spacing, electrostatic alignment, and dipole corrections [17,77,97,98,99,100,101].

Electronic descriptors such as DOS, PDOS, and HOMO–LUMO or band gaps are used to identify curvature-induced electronic states, apex-localized frontier orbitals, and changes in the electronic structure after adsorption or chemical modification [14,16,17,18,50,51,52,74,75,102,103,104,105]. Diffusion barriers provide information on ion mobility and are particularly relevant for alkali-ion storage and capacitive/electrochemical applications [21,22]. Optical absorption and related excited-state descriptors are useful for evaluating optoelectronic and nonlinear optical responses in nanocone-based systems [28,49]. Finally, elastic constants and related mechanical descriptors are required to assess the structural robustness, deformation response, and possible probe-tip applications [19,61].

### 5.3. Critical Evaluation of Computational Methods

Computational studies of carbon nanocones have employed a range of electronic structure and atomistic modeling approaches, including plane-wave DFT, localized-basis quantum-chemical calculations, semiempirical or tight-binding-type methods, classical force fields, and emerging machine-learning interatomic potentials. These approaches often differ in terms of the exchange–correlation functional, basis set, dispersion correction, model size, charge state, edge-passivation scheme, adsorption reference state, training-data quality, and treatment of periodic boundary conditions. As a result, direct comparison between different CNC studies is not straightforward, and reported adsorption energies, band gaps, work functions, charge transfer descriptors, diffusion barriers, and optical responses may vary substantially, even for nominally similar systems [14,16,17,18,19,20,21,22,28,50,51,52,106,107,108,109,110,111].

Commonly used methodological foundations for plane-wave DFT workflows include PBE-type exchange–correlation functionals, PAW potentials, Brillouin zone integration schemes, iterative total energy minimization, vacuum level alignment, and dipole corrections for slab or finite-cluster models [77,78,79,80,81,82,83,84,85,86,87]. Classical force fields and machine-learning potentials can extend simulations to larger systems and longer time scales, but they do not automatically provide electronic descriptors such as work function, charge transfer, or DOS/PDOS unless specifically coupled to electronic structure calculations or trained and validated for the relevant property. The main methodological factors affecting the CNC predictions are summarized in Table 5.

Table 5 provides the computational reporting checklist. A numerical comparison is treated as matched only when the relevant structure, termination, charge, reference state, observable definition, and protocol coincide or are controlled within the same study. Unreported fields are recorded as unavailable rather than assumed equal. Source-specific numbers can be reported as the original authors’ predictions, but are not pooled or used to infer a universal hierarchy when these conditions are unmatched.

A descriptor also has a defined domain of applicability. A frontier-orbital gap of a finite cluster is not a periodic band gap; a cluster ionization-energy estimate is not automatically a slab work function; an isolated-atom adsorption energy is not an electrode voltage; and a nanohorn aggregate measurement is not the intrinsic property of one ideal cone. These distinctions govern the source-resolved comparisons below [14,18,22,50,51,52,97,98,99,100,101].

### 5.4. Evidence Assignment and Numerical Selection

Evidence classes are assigned to a specific claim, not to a paper as a whole. DE requires a measured property or demonstrated function on a characterized CNC-containing architecture matching that claim. The exact architecture is named, such as a probe, multilayer cone–particle adhesive, or hollow array. IE is a measurement on a related material or different endpoint, such as a nanohorn aggregate composite used to motivate an isolated-CNC claim. CP is a calculation, including DFT, molecular dynamics or finite-element prediction, even when the model is explicitly CNC-specific. An untested proposal is identified as a hypothesis, not assigned a validation score.

For numerical entries retained in this revision, a primary-source locator, units, model identity, and property meaning must be available. Missing method fields are stated explicitly. Within-study pairs are preferred; cross-study values are not pooled unless the relevant structural and methodological fields match. The retained source records and their limitations are listed in Table S1. Untraceable illustrative intervals, inferred midpoint values and unmatched five-angle grids are excluded. This is a targeted narrative synthesis, not an exhaustive systematic search or meta-analysis.

No criteria weights or scalar composite score are introduced. Supplementary Figure S1 is explicitly schematic; its values are not used to assign DE, IE, or CP or to rank materials.

### 5.5. Figure Preparation and AI Disclosure

**Figure 4:**
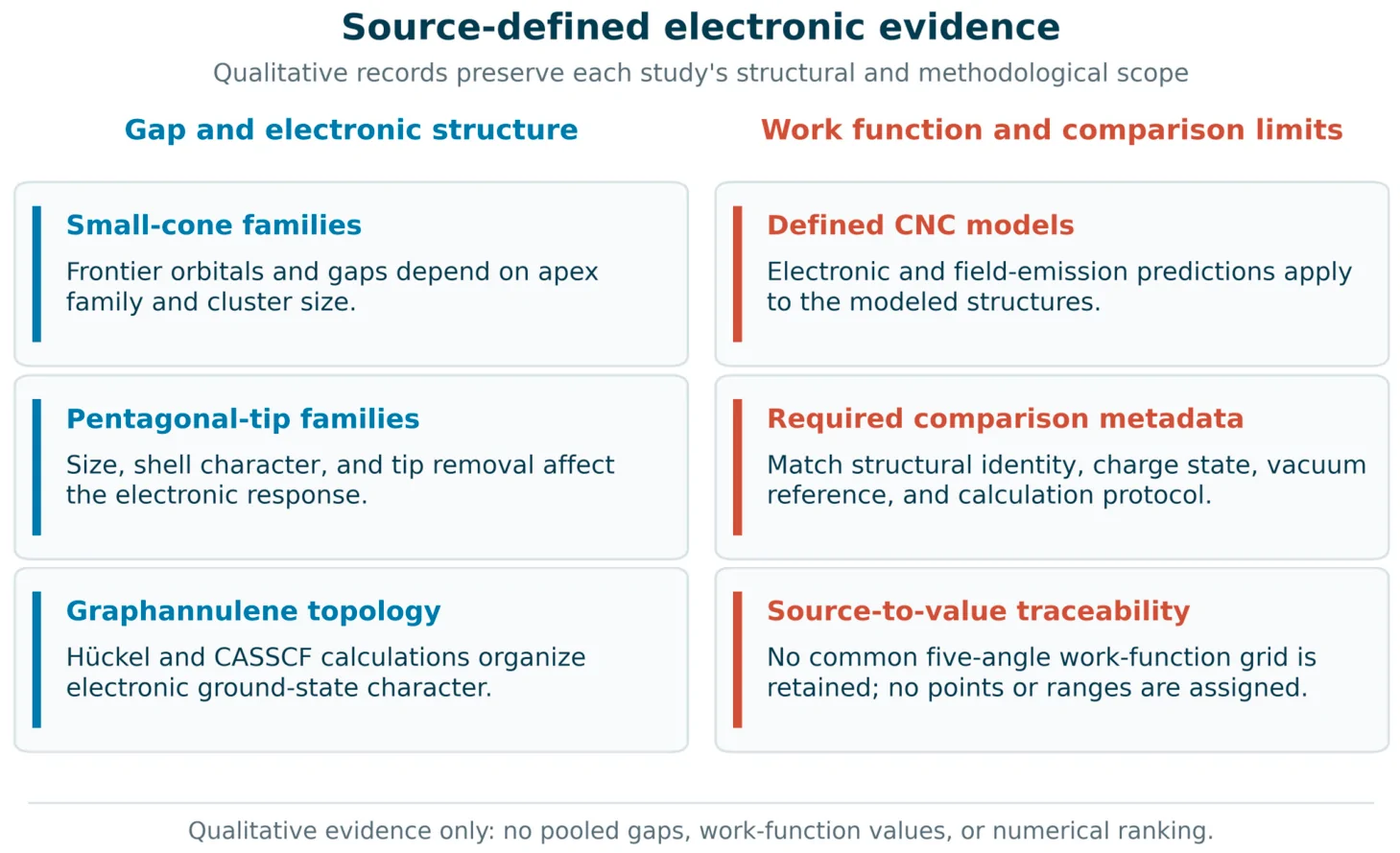
Source-defined electronic evidence in the original two-property visual format. Blue and red panels distinguish gap/electronic-state findings from work-function and emission-model limits. Equal-sized entries carry no numerical score, range, or ordering. The previous plotted intervals were compiled literature ranges, not statistical error bars; they are withdrawn because their per-value provenance was not reconstructable. This figure is a qualitative source map, not a five-angle numerical benchmark [17,50,51,52].

**Figure 5:**
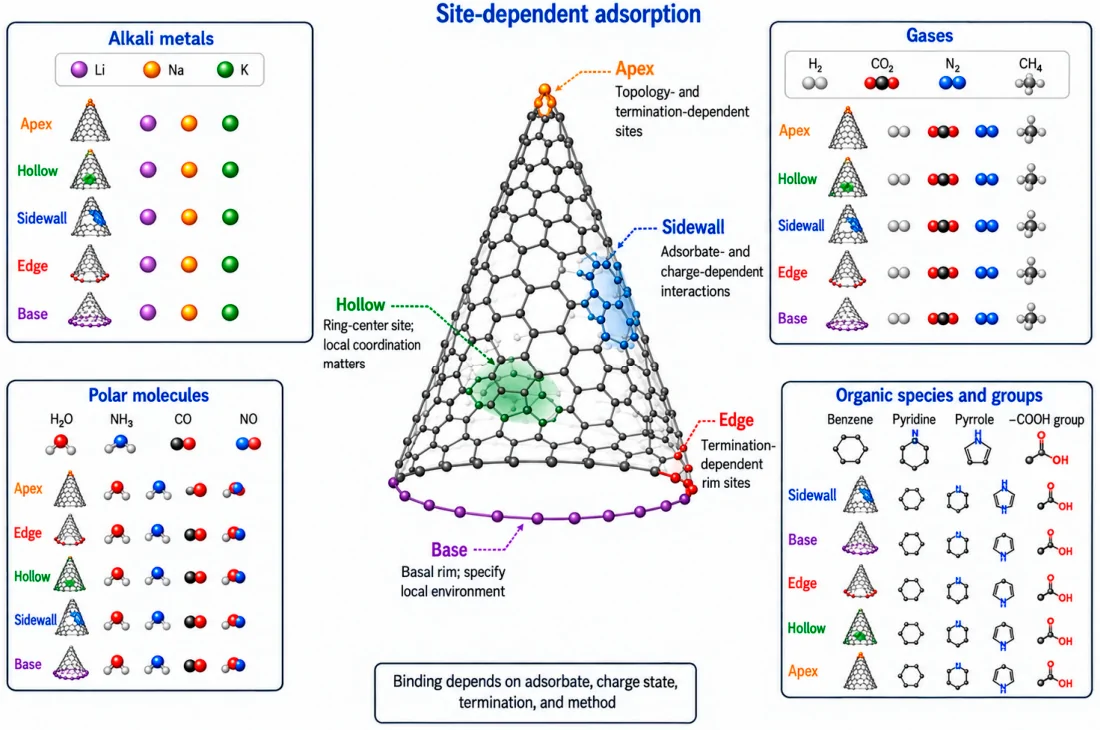
Site-dependent adsorption concepts with the original visual composition retained. Alkali metals, molecular species, and organic groups are shown alongside apex, hollow, sidewall, and rim categories. Species icons and colored regions are illustrative; they are not optimized adsorption structures or a prediction of relative binding strengths. Atomic-H chemisorption and molecular-H_2_ physisorption must be evaluated separately as explained in Section 9.2. AI-assisted conceptual illustration with the unsupported strength-ranking symbols removed [17,18,20,21,22,23,24,27,28,113,114].

**Figure 6:**
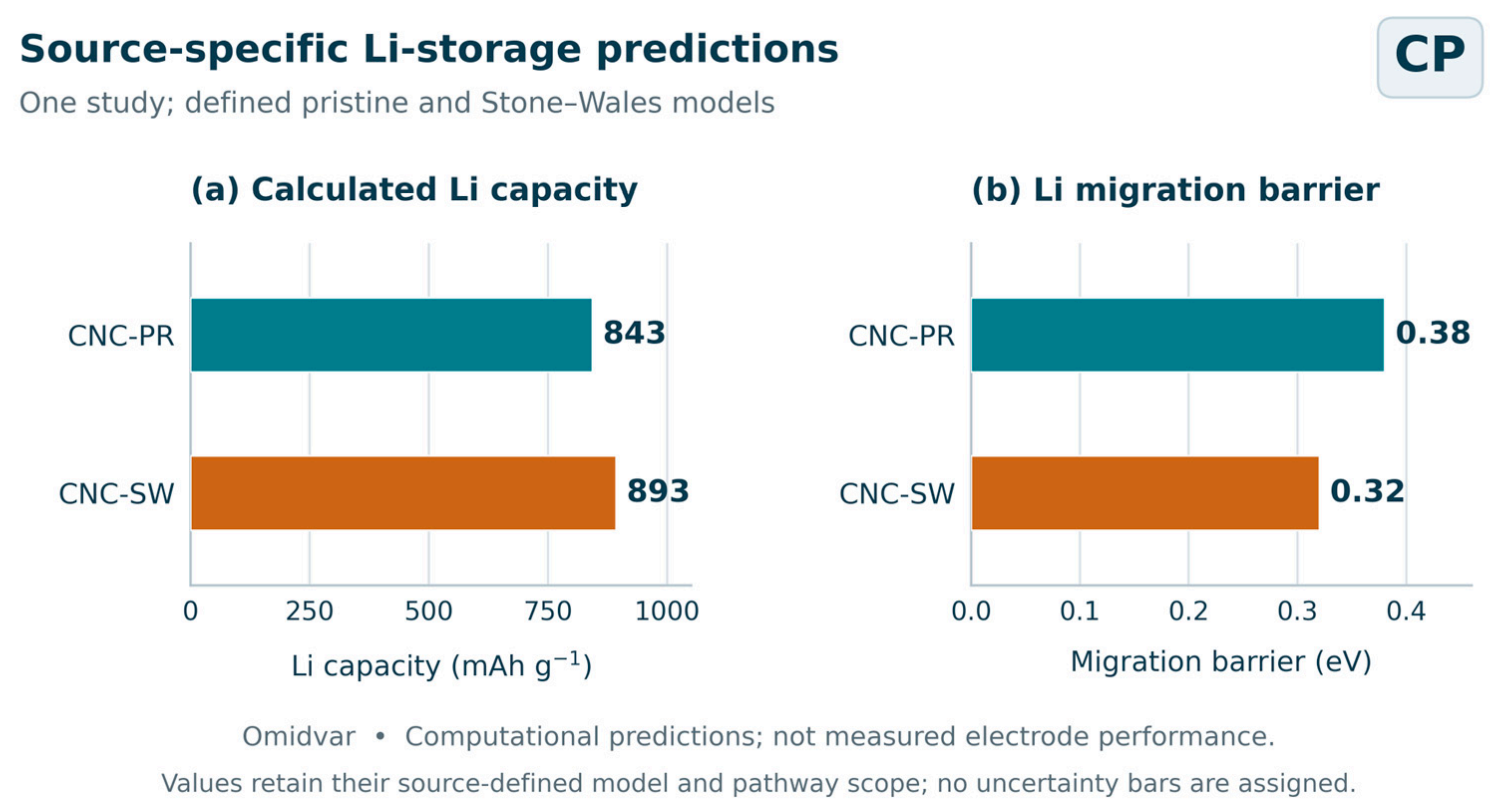
Source-specific Li-storage predictions from Omidvar [22], retaining a horizontal plotting format. Separate panels show calculated capacities of 843/893 mAh g^−1^ and migration barriers of 0.38/0.32 eV for CNC-PR/CNC-SW. These are reported point predictions from one study, not pooled adsorption-energy intervals or experimental measurements. No statistical uncertainty or literature-range bars are assigned. The former site-energy ranges are withdrawn; source and extraction limits appear in Table S1.

The submitted visual composition is retained where compatible with the evidence. Figure 3 remains the original AI-generated workflow illustration. Figure 4 is a coded qualitative source map. Figure 1, Figure 2 and Figure 5 and the graphical abstract receive limited AI-assisted image edits to clarify labels and remove unsupported curvature or binding/performance rankings. These conceptual graphics are not atomistic coordinate models, calculated charge densities, computed spectra, or experimental observations. The exact cone-angle relation is given independently in Equation (1). Figure 6 is a coded plot of the source-defined predictions in Table S1. The original radar is moved to Figure S1 and excluded from the analysis. Image files, prompts for the current edits, numerical inputs and plotting code are provided so the origin and role of each asset can be distinguished.

## 6. Geometry-Dependent Electronic Properties

### 6.1. Curvature-Induced Orbital Rehybridization

In flat graphene, the pz orbitals form a delocalized π-network. In CNCs, the conical curvature tilts these orbitals and produces σ–π rehybridization, the magnitude of which increases toward the apex [5,14,18,19]. This spatially non-uniform electronic perturbation distinguishes CNCs from CNTs, where the curvature is approximately uniform along the cylindrical wall, and from graphene, where curvature is absent in the ideal basal plane [4,5,7,115,116].

### 6.2. Apex-Localized Electronic States

Topological defects and broken sublattice symmetry can give rise to apex-localized electronic states in carbon nanocones (CNCs). Foundational studies have shown that the cone angle and defect topology control the low-energy electronic structure, including metallic or semiconducting behavior and localized states near the cone tip [14,16,17,18,74,102,103]. External electric fields can further modulate these low-energy states [102]. Recent studies on pentagonal nanocones have also indicated that apex topology, finite-size confinement, and aromaticity jointly influence the localization of frontier orbitals and optical transitions [50,51,52].

Additional theoretical work reinforces the conclusion that the electronic structure of nanocones is highly sensitive to topology, defect content, and cone architecture. Continuum and quantum-mechanical studies of single- and double-napped nanocones show that the local density of states near the apex can be strongly modified by the type of topological defect and may remain finite near the Fermi level for selected geometries [103]. Likewise, first-principles studies on double-walled carbon nanocones have indicated that interwall coupling, relative rotation, and apex separation can distort gap states and alter the electronic structure near the Fermi level [104]. These observations are consistent with the broader interpretation that apex topology and geometric coupling can serve as tuning parameters for electronic transport in conical carbon systems [103,104].

### 6.3. Source-Resolved Electronic Evidence

Table 6 separates the structural families and questions addressed by individual electronic-structure studies. Charlier and Rignanese linked apex resonances to the number and relative positions of pentagons [14]. Compernolle et al. compared small cones with triangular, square, and pentagonal apexes [50]. Mattoso et al. examined pentagonal graphannulene families, size, and charge effects [51], while Apriliyanto et al. used topological indices and electronic-state calculations [52]. These studies do not form a common-protocol numerical dataset spanning all five ideal cone angles.

Figure 4 retains the original electronic-descriptor theme and blue/red two-panel composition, but replaces untraceable numerical intervals with source-defined qualitative findings. The former 0.0–0.3 eV entry for n = 5 and the five-angle work-function grid are not retained because source-by-value model assignments could not be established. A near-zero finite-cluster gap is therefore not presented as demonstrated metallicity of every sharp cone.

For a specified small cluster, a near-zero gap can reflect edge-localized states, incomplete passivation, finite-size effects, electronic shell character, or the chosen functional. Distinguishing these possibilities requires size and termination series together with orbital localization and spin-state analysis. The present review does not attribute the withdrawn n = 5 interval to one artifact without a traceable originating calculation [18,50,51,52]. A general monotonic gap or work-function decrease across n = 1–5 is therefore not concluded.

Recent studies suggest that the electronic properties of carbon nanocones can be engineered not only by geometry but also by chemical modification. For example, mono-hydrogenation has been proposed as a route for bandgap engineering in nanocones, with calculated gap changes depending on the cone composition and hydrogen adsorption geometry [75]. Although these results were obtained for specific nanocone families, they support the broader conclusion that the CNC electronic structure is responsive to localized chemical perturbations, making nanocones attractive candidates for tunable nanoelectronic and optoelectronic applications [28,49,75].

In addition to static descriptors, such as the HOMO–LUMO gap and work function, geometry-dependent electronic functionality has also been predicted. Single-walled carbon nanocones with different cone angles have shown rectification behavior in DFT/NEGF calculations, with the 113° cone yielding the strongest diode-like response among the tested geometries [105]. This suggests that the cone angle may influence not only the local electronic structure but also the current–voltage asymmetry at the device level. However, these predictions await direct validation using structurally well-defined single-walled CNC devices [105].

## 7. Mechanical Properties and Stability

The mechanical stability of carbon nanocones (CNCs) reflects the interplay between strong sp^2^ bonding, curvature-induced strain, pentagonal defect topology, and edge or boundary effects of the CNCs. Sharper cones concentrate strain near the apex and may therefore exhibit higher local reactivity or reduced stability under extreme mechanical loading, whereas wider cones are expected to behave more similarly to finite graphene fragments because their curvature is lower and distributed over a larger surface area [19,61]. However, systematic mechanical datasets comparing all five ideal cone angles under identical simulation or experimental conditions are limited.

Graphitic bonding motivates mechanical studies of CNCs, but intrinsic cone mechanics, probe operation, and bulk polymer reinforcement are different endpoints. The CNC–polyethylene MD/finite-element study models an interphase contribution [65], whereas the AFM study validates a probe architecture [61]. Experimental nanohorn/PVA nanoindentation addresses a different filler, and measurement scale [67]. These distinctions are retained in the polymer discussion.

For mechanical benchmarking, graphene and CNTs provide useful reference systems for establishing sp^2^-carbon nanostructures. Monolayer graphene exhibits exceptional intrinsic stiffness and strength, whereas individual CNTs exhibit a very high Young’s modulus and characteristic bending and buckling behaviors [117,118,119]. These references are used here only as comparative benchmarks and not as direct evidence for CNC mechanical constants. Comparable systematic datasets for CNCs across the five discrete cone angles are still lacking, making it difficult to assign reliable values for Young’s modulus, buckling strain, fracture strength, or bending stiffness to ideal CNCs.

### 7.1. Mechanical Strain–Property Coupling

Mechanical strain can be used to tune the electronic and adsorption properties of curved sp^2^-carbon systems [120,121,122,123,124,125,126]. Because CNCs already contain intrinsic curvature-induced strain, external compression, tension, bending, or indentation may further modify orbital overlap, bond angles, local work function, adsorption energy, and transport behavior. Strain-engineered graphene and related curved carbon systems provide useful analogies for understanding these effects, although direct CNC-specific data are limited [120,121,122,123,124,125,126].

The strain-induced curvature effects reported for graphene and related systems provide a useful framework for interpreting the possible thermomechanical behaviors of CNCs. Curvature and strain can influence the electronic structure, phonon dynamics, and lattice thermal conductivity of graphene-based systems [120,121,122,123,124,125,126,127]. Recent theoretical studies have suggested that strain-induced curvature may couple mechanical deformation to electronic and transport properties, although this relationship has yet to be systematically verified for CNCs [127]. In particular, systematic computational studies comparing all five CNC geometries under identical strain protocols have not yet been reported.

### 7.2. Carbon Nanocone Probes for Polymer Characterization

CNC AFM probes have been used to characterize polymer films and composites [61]. The measured performance applies to the tested probe architecture and characterization task. Tip radius, attachment, stiffness, wear, and calibration must be evaluated for that task rather than inferred solely from an ideal cone angle.

In this application, the CNC is the probe material and the polymer is the specimen; the experiment therefore validates a characterization tool rather than a nanocone-filled polymer composite. Relating apex geometry and probe mechanical behavior to the measured polymer response is necessary when interpreting local nanomechanical maps [61]. The result provides a direct polymer-oriented use of CNC geometry, while broader claims about composite reinforcement require separate material-level evidence.

### 7.3. Critical Assessment

Matched mechanical datasets for isolated, angle-controlled CNCs remain limited. Calculated elastic or buckling descriptors depend on geometry, potential, boundary conditions, and loading protocol; composite reinforcement additionally depends on dispersion, interface structure, filler orientation, and loading fraction [19,61,65]. Section 9.5 distinguishes those predictions from measured nanohorn–polymer properties.

Therefore, the reported CNC mechanical values should be treated cautiously unless the model size, cone angle, force field, boundary conditions, edge passivation, loading direction, and loading protocol are explicitly stated. Future studies should prioritize standardized molecular dynamics and first-principles calculations for n = 1–5 CNCs, followed by experimental nanoindentation or in situ SEM–AFM measurements on structurally characterized cone-rich materials. This is important because mechanical stability directly affects the performance of CNCs in field-emission, scanning-probe, flexible-electrode, and mechanically loaded sensing-platform applications.

## 8. Thermal Evidence and Its Limits

### Computational Results and Experimental Gap

No direct experimental measurement of intrinsic thermal transport or thermal rectification in isolated, angle-controlled CNCs was identified in the literature evaluated here. CNC-specific computational results do exist: Yang, Zhang, and Li used molecular dynamics to predict thermal rectification over 200–400 K [128]. This is model-specific computational evidence, not a measured CNC thermal diode. The previous treatment of all CNC rectification as an unstudied hypothesis was therefore too broad.

Graphene and SWCNT measurements provide context rather than transferable CNC constants. Balandin et al. reported room-temperature Raman-based conductivity estimates for suspended single-layer graphene [129]; Pop et al. studied a suspended metallic SWCNT of specified length and diameter [130]. Section 14 provides contextual benchmarks for these measurements. They cannot be assigned to CNC aggregates, powders, films, or polymer composites, where porosity, orientation, interparticle contacts and interfaces alter the measured effective conductivity.

Polymer-associated thermal outcomes also require a defined objective. PVA/phytic-acid/nanohorn aerogels target insulation [68], PANI/nanohorn formulations couple electrical and thermoelectric response [69], and nanocarbon-containing responsive gels convert optical absorption into a polymer transition [70]. These are measured composite or gel functions, not evidence that an isolated CNC has the thermal conductivity of graphene or a nanotube. Thermal focusing and interface-resistance control remain application-specific questions requiring matched simulations and measurements.

## 9. Adsorption and Surface Reactivity

### 9.1. Site-Dependent Adsorption

One of the most functionally important characteristics of carbon nanocones (CNCs) is their pronounced, site-dependent adsorption behavior. Because the local curvature, coordination environment, edge termination, and electronic localization vary substantially from the apex to the base region, different parts of the same nanocone may exhibit distinct adsorption energies, preferred binding geometries, and charge-transfer characteristics [17,18,20,21,22,23,24]. The main adsorption-relevant regions of CNCs and their expected functional roles are summarized in Table 7.

As summarized in Table 7, the apex region is generally predicted to be among the most reactive parts of the nanocone because it combines a strong local curvature, topological defects, and localized electronic states. Hollow-type coordination environments may provide favorable multicarbon interaction sites for alkali-metal adsorption and ion diffusion, whereas sidewall regions offer comparatively graphitic adsorption environments that are more relevant to molecular interactions and charge transport. In contrast, the base and edge regions can dominate the local reactivity when dangling bonds, partial passivation, oxidation, or functional groups are present. Consequently, adsorption studies on CNCs should explicitly specify the adsorption site, edge termination, reference-state convention, optimized geometry, charge state, and computational methodology to enable meaningful comparisons between different studies.

Figure 5 preserves the original cone, colored site categories and surrounding adsorbate panels. The earlier greater-than symbols and weak/moderate/strong legend have been removed because they implied a universal binding hierarchy. Site effects depend on the adsorbate, charge, termination, reference state, and computational method; the drawing assigns no generic apex, sidewall, hollow, or rim binding rank.

### 9.2. Source-Resolved Adsorption Evidence

Table 8 records what the cited adsorption studies actually model. The former Li/Na/K apex, sidewall, hollow, and edge intervals were withdrawn because per-value source assignments and compatible reference states could not be reconstructed. Consequently, no pooled site-energy ordering or apex-to-sidewall energy difference is inferred from those intervals.

Omidvar studied pristine and Stone–Wales-defected CNC anode models for Li storage [22]. Hsu et al. studied perfect and defective cone-shaped carbon models for Na/K storage [21]. These are study-specific model comparisons, not a single Li/Na/K benchmark. Their reported adsorption quantities should not be combined without matching structural, charge, and reference-state conventions.

Hydrogen requires a separate chemical classification. The cited vacancy-engineered CNC study [114] concerns a single atomic H, using finite H-terminated models and B3LYP/6-31G; it does not establish the earlier weak H_2_ physisorption intervals. The companion hydrogenation work [113] uses a multi-H, atomic-reference energy convention. Neither can be treated as an eV-per-H_2_ physisorption dataset.

For molecular H_2_ and other weakly interacting neutral molecules, dispersion, zero-point energy, temperature, pressure, and coverage should be evaluated in the appropriate molecular-adsorption calculation [88,89,90,91,92,93]. Such physisorption is discussed separately from alkali-metal binding and atomic-H chemisorption. No new molecular-H_2_ numerical range is assigned in this review.

For any reported adsorption interval, the convention must run from the lower numerical value to the higher value, so negative-energy limits are written from more negative to less negative. The revision removes the unsupported reversed intervals rather than making their ordering appear to validate their provenance.

### 9.3. Effect of Doping and Functionalization

Doping and functionalization can substantially modify the adsorption strength, charge transfer, and selectivity of CNCs. Heteroatom doping, metal decoration, vacancy engineering, and surface functionalization can introduce localized electronic states and stronger binding centers that are absent in pristine CNCs. For example, N-doped and Pt-decorated CNCs have been proposed for CO_2_ hydrogenation to produce formic acid [33]. TiO_2_-, B-, and N-modified CNC-like systems have been investigated for changes in their electronic, linear optical, and nonlinear optical properties [28]. Zn-doped CNCs have also been studied as molecular carriers for drug delivery-related adsorption [27]. In gas-sensing contexts, functionalized, decorated, or doped nanocone strategies have been proposed to improve molecular detection performance relative to pristine CNCs [24]. These examples indicate that functionalization is likely essential for translating the intrinsic site selectivity of CNCs into practical sensing, catalytic, biomedical, and optoelectronic applications [24,27,28,33].

### 9.4. Critical Assessment of Adsorption Data

Several caveats should be considered when interpreting compiled adsorption data. First, cross-study consistency remains limited. Differences in the model size, cone angle, edge treatment, charge state, exchange–correlation functional, dispersion correction, and adsorption-reference state can produce noticeably different Eads values for nominally similar systems [14,16,17,18,19,20,21,22,28,50,51,52,88,89,90,91,92,93]. Currently, few studies use identical CNC models and computational settings, making direct numerical comparisons difficult.

Adsorption records are interpreted at the conditions actually calculated. Electronic total-energy differences for isolated optimized configurations do not directly establish finite-temperature selectivity, recovery time, cycling stability, or biological release. Those claims require the relevant free-energy corrections and application-specific validation. Table 8 therefore separates chemical identities and model scopes without converting heterogeneous electronic energies into performance metrics.

### 9.5. Polymer Matrices Interfaces and Conductive Composites

CNC–polymer studies extend beyond metrology and drug-scaffold models. Taheri and Fakhrabadi combined molecular dynamics with finite-element analysis for CNC-reinforced polyethylene [65]. The simulations distinguish polymer interphases inside and outside the cone; the denser inner region and its geometry dependence affect the modeled effective elastic response. This is a CNC-specific computational interphase result. It does not independently establish experimentally measured reinforcement, and the interphase constitutive assignment and simulated morphology remain part of the model.

Experimentally, Knaapila et al. assembled conducting cone–particle pathways in a commercial two-component adhesive using an AC electric field [64]. Conductivity was directional and persisted after curing. The tested material consists of cone–particle assemblies, rather than isolated monodisperse single-walled CNCs. This experiment establishes an actual processing–network–conductivity connection while also showing why filler concentration, orientation, cure history, and interparticle contacts must accompany any intrinsic-curvature descriptor.

Mechanical and thermal polymer outcomes must be separated. Kadambi et al. measured nanoindentation modulus and hardness in PVA/SWNH composites; their increases at low filler content concern local indentation response, not tensile strength of ideal-CNC composites [67]. Xu et al. combined nanohorns and phytic acid in porous PVA aerogels to obtain mechanical resistance, flame-retardancy, and insulation functions [68]. The porosity and co-additive are part of that architecture. Low effective conductivity in an aerogel and enhanced heat conduction in a dense filler network are different design objectives.

Conductive polymers and inert polymer matrices also involve different network mechanisms. Famengo et al. mixed SWCNHs with doped PANI and evaluated electrical, Seebeck, and thermal responses [69]. Montes et al. adjusted the nanohorn fraction in epoxy carbon-paste electrodes to tune the electroactive network [71]. In oxidized-SWCNH/PVP humidity films, Serban et al. showed that electrode spacing affects the apparent percolation threshold [72]. These experiments establish formulation- and geometry-dependent behavior; an apparent threshold is not an intrinsic constant of a cone tip.

Miyako et al. used nanocarbons, including SWNHs, in agarose and PNIPAM gels and observed near-infrared-driven polymer transitions [70]. That supports optical-to-thermal coupling in a responsive gel. It does not demonstrate therapeutic release or cytocompatibility. Separately, the DFT study of chitosan-modified CNC scaffolds assesses ciclopirox binding and electronic descriptors [66]; experimental formulation stability, release kinetics, and biological outcomes remain separate validation tasks.

Across these cases, dispersion and interface descriptors should include the filler architecture and oxidation, aggregate size, accessible cone interior, polymer chemistry, filler fraction, orientation, interparticle contacts, and processing history. Bulk or device claims then require measurements at matched temperature, loading, and geometry. General polymer/organic energy-level-alignment studies [131,132] provide interface context, while the source-defined CNC and nanohorn studies above establish the actual evidence. Table S2 records the material and evidence scope of each added study.

## 10. Energy-Storage Applications

### 10.1. Li-Ion Storage

Carbon cone-based materials are promising candidates for electrochemical energy-storage studies because they combine graphitic conductivity, defect-rich surfaces, curvature-induced charge redistribution, and diverse adsorption sites [13,21,22]. In Li-ion systems, Li can bind to hollow, sidewall, edge, and apex sites with different strengths. A practically useful anode should combine sufficiently favorable adsorption, low migration barriers, high electronic conductivity, and structural reversibility. Very weak adsorption may limit storage, whereas excessively strong adsorption may cause irreversible trapping and poor rate capability.

Pristine and Stone–Wales-defected carbon nanocones have been modeled as Li-storage anodes [22]. The predicted defect-dependent storage and migration behavior concerns those defined computational models; it is not experimental electrode performance. Direct experimental battery studies on well-characterized isolated CNC electrodes remain a separate validation task.

### 10.2. Na- and K-Ion Storage

Na^+^ and K^+^ ions are larger than Li^+^ and often require more open, curved, or defect-rich adsorption environments than Li^+^ does. Carbon cone-shaped anodes have been theoretically proposed for Na- and K-ion batteries, where curvature-driven charge redistribution and site diversity may enhance ion adsorption [21]. However, Na- and K-ion storage in isolated CNC models remains computationally predicted. Future studies should evaluate not only adsorption energies but also diffusion barriers, ion clustering, voltage profiles, structural expansion, charge/discharge reversibility, and cycling stability.

The broader alkali-ion storage relevance of cone-rich or defect-rich carbon architectures can also be connected to hard-carbon and nanohorn-based systems; however, these materials should not be treated as direct experimental validation of ideal atomistic CNC models [25,26,54,55,56,57].

### 10.3. Capacitive Electrochemical Applications and Deionization

Cone-rich porous carbons, hollow carbon nanocone arrays, and nanohorn-containing networks can support capacitive or electrochemical ion-storage processes by increasing the accessible surface area, ion accessibility, and charge accumulation [54,55,56,57,62]. However, the available experimental evidence should be interpreted with caution. Hollow carbon nanocone arrays grown on carbon fiber cloth have been demonstrated as free-standing electrodes for capacitive deionization, which is related to capacitive ion storage but should not be overgeneralized as direct proof of supercapacitor performance [62]. This represents one of the clearest experimental examples of a cone-based carbon architecture in an electrochemical application; however, it should be distinguished from isolated, atomistically defined single-walled CNCs.

Indirect experimental support for cone-rich carbon architectures in electrochemical storage is provided by carbon nanohorn systems. For example, bud-type carbon nanohorns with tip openings induced by mild oxidation have been reported to exhibit an enhanced surface area, promising high-pressure CO_2_ adsorption, and Li-ion battery performance comparable to that of carbon nanotubes and carbon nanofibers [25]. These results are important because they demonstrate that conical graphitic motifs can contribute meaningfully to the adsorption and electrochemical behavior of real materials [25]. However, such findings should still be interpreted as evidence for nanohorn aggregate materials rather than direct validation of ideal, isolated carbon nanocone models [25,54,55,56,57].

### 10.4. Quantitative Benchmarking: CNCs vs. Other Carbon Anodes

Table 9 compares source-defined ion-storage claims rather than generic material-wide energy ranges. A conventional graphite LiC_6_ capacity is a stoichiometric reference [133]. Figure 6 retains the earlier horizontal plotting style but displays the CNC-PR/CNC-SW capacities and migration barriers reported by Omidvar for two models in one study [22]. Nanohorn and hard-carbon electrodes provide experimental context for their own architectures [25,26]; they do not experimentally validate the calculated CNC capacities.

Omidvar reports Li capacities of 843 and 893 mAh g^−1^ and migration barriers of 0.38 and 0.32 eV for CNC-PR and CNC-SW, respectively, with a reported CNC-SW open-circuit voltage of 0.59 V [22]. Figure 6 plots the capacities and barriers on separate axes. These are source-specific computational predictions bounded by the structures, loading assumptions and protocol; they are not measured reversible capacities or experimental migration barriers of a CNC electrode.

Strong isolated binding alone establishes neither reversible storage nor irreversible trapping. Metal chemical potentials, loading, aggregation, voltage, diffusion pathways, electrolyte interactions, and structural changes must be evaluated. No universal −3.0 eV trapping threshold or apex-specific performance advantage is adopted. The next discriminating test is a structurally characterized cone-containing electrode with matched loading and cycling controls, or a calculation that reproduces those conditions.

A comparison with hard-carbon systems is particularly instructive for Na- and K-ion storage. Recent template-derived hard carbons have achieved high reversible capacities in both Na and K cells, indicating that structurally disordered but experimentally mature carbon frameworks currently provide a stronger practical basis for alkali-ion storage than hypothetical CNC electrodes [26]. From this perspective, CNCs are better viewed as mechanistic model systems for curvature-enhanced adsorption and site-selective ion binding, whereas hard carbons remain the more advanced and application-relevant benchmark for real Na/K battery anodes [26].

## 11. Sensing Applications

### 11.1. Transduction Mechanisms

Kumar et al. calculated ClCN adsorption and electronic-gap changes for pristine and chemically modified nanocones, including Al- and Ga-substituted models [24]. Their results support a target- and modification-specific electronic-response hypothesis; they are not measurements of conductance, selectivity, or device recovery. Rajabi et al. used DFT to evaluate lomustine adsorption on a nanocone in a carrier/sensor context [23]. That evidence concerns modeled binding and electronic descriptors rather than an experimentally calibrated detection limit.

A proposed work-function signal also needs a device-level test. The CNC electronic and field-emission calculations [17] and broader nanocarbon work-function studies [97,98,99,100,101] motivate vacuum-level or contact-potential measurements, but do not demonstrate six interchangeable CNC-sensing mechanisms. Any transduction claim must identify the actual observable, target species, contacts, and environmental conditions.

### 11.2. Selectivity Challenge

Pristine carbon surfaces generally interact weakly with many gas molecules, and their sensing response often relies on small adsorption-induced changes in charge transfer or electronic structure [2,5,8,134]. Doped, decorated, or functionalized CNCs can introduce selective binding centers; however, practical sensors require a balanced adsorption energy that is strong enough for detection and weak enough for recovery. For practical deployment, humidity tolerance, operating temperature, reversible adsorption–desorption behavior, and long-term stability must be addressed [20,23,24,27,28].

Recent DFT evidence supports the broader conclusion that pristine carbon nanocones are often insufficient for practical gas sensing and that chemical modification is likely necessary to obtain strong and selective responses. In a study on ClCN detection, pristine CNCs produced only limited electronic perturbations, whereas Al- and Ga-doped CNCs showed stronger adsorption and larger changes in the electronic gap [24]. These results are consistent with the general sensing framework outlined here: apex curvature alone may enhance reactivity relative to graphene-like surfaces, but dopants, decorations, or functional groups are typically required to translate the intrinsic sensitivity into a practically measurable electronic signal [24].

### 11.3. Comparative Sensing Descriptors

Table 10 gives source-specific examples rather than unverified gas-by-gas ranges for generic graphene, CNT, and CNC platforms. A DFT adsorption response, an experimental polymer/nanohorn humidity response and an operating isolated-CNC sensor are distinct evidence claims. The material and measured or calculated endpoint are therefore stated in each row.

This comparison strengthens the rationale for distinguishing between pristine and modified CNC sensing platforms. A single adsorption-energy window or work-function shift is unlikely to describe all sensing scenarios. Instead, the useful design space probably involves a combination of the cone angle, apex accessibility, dopant identity, dopant density, target molecule, adsorption strength, charge-transfer response, and recovery kinetics [23,24,27,28]. Accordingly, future CNC sensing studies should move beyond single-adsorbate proof-of-concept calculations and compare pristine, doped, decorated, and functionalized nanocones under consistent computational conditions [24].

The polymer/nanohorn example illustrates a practical transduction variable that isolated adsorption energies omit. In oxidized-SWCNH/PVP humidity films, polymer swelling and conductive-network connectivity contribute to resistance changes, and the observed percolation threshold changes with electrode spacing [72]. This is indirect experimental evidence for prospective CNC devices, not a curvature-only mechanism.

The CNC-specific studies considered here do not supply a directly validated gas-sensing device based on isolated, angle-controlled nanocones. Future tests should compare matched pristine and modified materials, specify target concentration and humidity, and measure reversibility, selectivity, drift, and recovery. Computational binding and electronic shifts remain screening descriptors until linked to those observations.

## 12. Optical Response and Field Emission

### 12.1. Optical Response

The optical response of carbon nanocones (CNCs) is governed by their electronic structure, π–π* transitions, curvature, finite-size effects, and chemical functionalization. Because CNC-specific optical datasets remain limited, most interpretations currently rely on broader graphene, graphite, and CNT optical benchmarks, together with recent CNC-like or nanocone-based theoretical studies [28,39,40,41,42,43,44,45,46,47,48,49,135,136,137,138,139,140,141,142,143,144,145,146,147,148]. The relevant descriptors include the dielectric function, absorption coefficient, reflectivity, refractive index, and energy-loss function. Curvature and topological defects can shift the oscillator strength, introduce low-energy transitions, and modify light–matter interactions relative to flat graphene or cylindrical CNTs.

For a rigorous interpretation, independent-particle spectra must be distinguished from many-body GW and excitonic BSE treatments. Studies on graphite, graphene, and CNTs have shown that anisotropy, depolarization, excitons, and plasmons can substantially alter the calculated optical spectra [39,40,41,42,43,44,45,46,47,48,135,136,137,138,139,140,141,142,143,144,145,146,147,148]. CNC-specific optical studies remain less developed; however, TiO_2_-, B-, and N-modified CNC-like systems and Au_2_–CNC structures indicate that chemical modification, curvature, and external fields can strongly affect optical absorption and nonlinear optical responses [28,49]. Therefore, optical claims for CNCs should be presented as geometry- and model-dependent predictions unless supported by experimentally validated CNC optoelectronic devices.

### 12.2. Field Emission

Field emission is a natural application for CNCs because a sharp apex can enhance the local electric field and reduce the effective emission barrier [17,29,30,31]. The performance of a CNC-based field emitter depends on the apex radius, work function, conductivity, structural stability, alignment, emitter density, and electrostatic screening between neighboring emitters.

Experimental evidence for field-emission functionality already exists at the level of carbon-based nanocone films. Graphitic nanocone films have been reported to exhibit low turn-on fields and high emission currents, whereas mixed diamond/graphitic nanocone films exhibit similar strong emissions with improved stability [29]. These results do not directly validate the isolated single-walled CNC models with well-defined apex topologies; however, they provide device-level support for the broader idea that cone-shaped carbon architectures are favorable for field emission because of their sharp geometry and conductive carbon framework [29].

Fowler–Nordheim-type analysis relates field emission to the local electric field, work function, and field enhancement factor [30,31]. A smaller apex radius can increase local field enhancement, but the effect must be evaluated for a defined emitter shape, spacing, and electrostatic boundary condition; the ideal cone angle alone does not fix the apex radius or enhancement factor [30,31]. For a defined CNC model, a reduction in work function can lower the emission barrier. Absolute values depend on cone size, edge termination, surface dipoles, vacuum alignment, and computational setup [17,97,98,99,100,101,131,132]. Field-emission comparisons must distinguish geometry-driven field enhancement from intrinsic work-function shifts.

### 12.3. Validation Status

Field-emission analysis must distinguish geometry-driven field enhancement from intrinsic work-function changes. Experimental field emission has been demonstrated for multi-walled carbon nanocone structures and cone-rich carbon films, but not yet for isolated single-walled CNCs with precisely defined cone angles and apex topologies [29]. Thus, existing experiments validate cone-rich carbon emitters at the film/device level, whereas field emission from isolated, angle-controlled CNCs remains primarily a computational or conceptual prediction.

In addition to field emission, carbon nanocones have been proposed as nanoelectronic rectification elements. DFT-based transport calculations predicted cone-angle-dependent rectifying behavior in single-walled CNCs, suggesting that conical asymmetry may be exploited not only for enhanced local fields but also for directional charge transport [105]. Together, these studies suggest that CNCs may be technologically relevant for field-emission and nanoelectronic device concepts, although most device-level proposals remain computational and have not yet been demonstrated using isolated, angle-controlled nanocones [29,105].

## 13. Catalytic Applications

### 13.1. Curvature and Pentagon Defects in Carbon Catalysis

Curved and pentagon-enriched sp^2^-carbon sites can provide coordination-diverse environments for catalytic centers [34,35,36,37,38,149]. Compared with flat graphene-like basal planes, curved carbon frameworks can modify the local coordination, charge distribution, metal–support interactions, adsorption geometry, and frontier-state localization. Recent studies suggest that topology and curvature may serve as useful activity descriptors for several catalytic processes, although direct experimental evidence for structurally well-defined CNC catalysts remains limited [34,35,36,37,38,149].

Pentagon defects and local curvature are particularly relevant to CNCs because the cone apex is defined by a nonhexagonal topology and curvature concentration [11,12,13,14,15,16,34]. This makes CNCs a useful model system for examining how apex topology, defect density, dopant configuration, and single-atom coordination may influence catalytic activity. However, most studies cited in the CNC context do not experimentally investigate isolated angle-controlled CNCs. Instead, they typically examine pentagon-rich disordered carbons, warped graphene, N-doped curved carbons, curvature-rich catalyst supports, and related nanostructured carbon systems. Therefore, these studies should be treated as mechanistic analogues rather than direct validations of ideal CNC catalysts.

### 13.2. CNC-Specific Versus Indirect Catalytic Evidence

Catalytic evidence is classified by both study method and material identity:

CNC-specific computational prediction: calculations on explicitly defined CNC-supported catalytic sites, including the systems in refs. [20,33]. These are CP records, even though the model is directly CNC-specific.

Indirect experimental or computational context: curved graphene, warped carbon, N-doped curved carbon, and curvature-rich single-atom catalyst supports [35,36,37,38,149]. The study method must be stated for each claim.

Pentagon-defect context: pentagon-rich or disordered carbons support mechanisms associated with non-hexagonal sites, but do not establish the same outcome for an isolated CNC [34,37].

A direct experimental CNC claim requires a measured catalytic endpoint on a characterized CNC-containing architecture. Model specificity alone does not qualify as experimental validation. Table 11 retains the architecture and method of each cited catalytic study.

### 13.3. Critical Assessment

To move CNC catalysis beyond conceptual and computational predictions, future studies should compare identical catalytic reactions across n = 1–5 nanocones, quantify the adsorption energies and activation barriers for key intermediates, and separate apex-topology effects from dopant, vacancy, edge, and metal-cluster effects. Experimental studies should also examine the closest available proxies, such as controlled-curvature N-doped carbons, SWCNH aggregates, cone-rich carbon electrodes, or pentagon-rich carbon frameworks, while clearly stating that these materials provide indirect rather than direct CNC validation results.

## 14. Contextual Benchmarks and Comparison Limits

Table 12 places source-defined CNC results beside contextual graphene, nanotube and polymer-composite evidence. It does not identify an intrinsically superior material. Geometry-controlled computational effects and experimentally obtained device or composite responses answer different questions and must retain their material, method, and measurement scope.

A small gap in one finite cone, a favorable binding energy and a high composite conductivity cannot be combined into a common performance scale. The descriptors have different units, reference states and material domains. A useful comparison first fixes the intended function and then matches the observable and measurement or simulation conditions.

Supplementary Figure S1 and Table S3. contain only illustrative schematic scores. No empirical calibration, reproducible scoring rubric, weighting or evidence-based ranking is claimed. These scores are excluded from the descriptor framework, the evidence classification and the conclusions.

The main limitation is the incomplete connection between precisely defined CNC models and scalable, well-characterized material architectures. Existing cone–particle adhesives, nanocone arrays, and probe structures demonstrate particular functions [61,62,64], while nanohorn–polymer composites demonstrate aggregate-level functions [67,68,69,70,71,72]. Neither category licenses a universal property assignment to every ideal cone angle.

Accordingly, benchmarking should preserve the target architecture, property definition, operating conditions, and evidence class. Calculations can isolate mechanisms; experiments can validate a specified material or device. Their connection requires structural correspondence and an endpoint that tests the claimed mechanism.

## 15. Challenges and Future Perspectives

### 15.1. Standardization of Carbon-Nanocone Models

The reporting fields and comparability criteria are consolidated in Section 5.2, Section 5.3 and Section 5.4 and Table 5. The next priority is a shared set of reference structures with published coordinates and convergence data, so independent groups can reproduce defined comparisons rather than add further unmatched descriptor intervals.

### 15.2. Bridging Theory and Experiment

The second challenge is the gap between the idealized theoretical models and the experimentally produced cone-rich carbon materials. Most DFT studies assume clean, isolated, atomistically defined CNCs with known apex topologies and edge terminations. In contrast, experimental samples are typically heterogeneous and may contain pores, oxygen-containing groups, residual catalysts, amorphous carbon, graphitic fragments, nanohorns, nanodiscs, and multi-walled cone-like structures [11,12,13,53,58,59].

Bridging this gap requires multiscale modeling that uses experimental microscopy, spectroscopy, and diffraction data as structural inputs. Ab initio molecular dynamics can be used to examine realistic surface reconstruction, oxygenation, and adsorbate mobility. In addition, machine-learning interatomic potentials trained on DFT data may enable the simulation of larger cone-rich aggregates, SWCNH-like assemblies, and electrode-scale porous networks that cannot be treated directly using standard first-principles calculations [108,109,110,111].

### 15.3. Realistic Operating Environments

Most computational studies on CNC adsorption, sensing, catalysis, and ion storage have been performed under idealized vacuum or isolated-adsorbate conditions [20,21,22,28,33]. However, practical battery, capacitive, sensing, and catalytic devices operate in more complex environments involving electrolytes, humidity, applied bias, temperature, finite surface coverage, and competing adsorbates. These factors may alter the adsorption energies, charge transfer, work function shifts, diffusion barriers, and reaction pathways, indicating that vacuum-phase descriptors should not be interpreted as direct device-level performance metrics.

Therefore, future studies should move beyond single-adsorbate vacuum calculations and, where appropriate, consider more realistic operating conditions, such as solvent or electrolyte environments, finite adsorbate coverage, applied electrochemical potential, temperature effects, and operando-like structural models. This is particularly important for Li/Na/K storage and gas sensing, where isolated adsorption energies alone are insufficient to predict the cycling behavior, reversibility, recovery kinetics, selectivity, or long-term stability. Currently, the gap between idealized DFT predictions and realistic operating conditions remains one of the largest barriers to evaluating CNCs as practical materials.

### 15.4. Controlled Synthesis

The most critical experimental limitation is the absence of reliable synthesis methods for isolated CNCs with a single predetermined cone angle, defined apex topology, controlled edge termination, sufficient production yield, and minimal co-produced impurities in the same batch. Although graphitic cones and carbon nanocones have been observed experimentally, the products usually contain mixtures of cone angles, nanodiscs, graphitic fragments, amorphous carbon, nanohorns, and multiwalled structures [11,12,13,58,59,63].

Controlled synthesis is essential because many predicted CNC advantages depend on the precise apex geometry. For example, field emission, Li adsorption, work function reduction, and localized electronic states are all expected to vary significantly with the cone angle and apex defect pattern [14,16,17,18,21,22]. Bottom-up molecular synthesis, template-directed CVD, plasma-assisted routes, and post-synthetic separation strategies are promising directions; however, the routine production of monodisperse, angle-selected CNCs suitable for device integration has not yet been demonstrated at the material scale [13,63].

### 15.5. Machine Learning and High-Throughput Screening

High-throughput computation and machine learning can accelerate CNC design by systematically mapping a multidimensional descriptor space that includes the cone angle, number of pentagonal defects, dopant type, vacancy concentration, adsorption site, adsorbate identity, surface functionalization, charge transfer, work function, and diffusion barrier [20,21,28,33,34,35,36,37,38,108,109,110,111,149]. Such approaches are especially attractive for screening CNCs in ion storage, gas sensing, field emission, and catalysis, where many combinations of dopants, adsorbates, and surface sites are possible [20,21,28,33,34,35,36,37,38,149].

Machine-learning interatomic potentials and data-driven atomistic models have already demonstrated that DFT-level simulations can be accelerated for molecular, graphene-like, and carbon-rich systems. Neural network potentials, Gaussian approximation potentials, active learning force fields, and equivariant graph neural-network models provide relevant methodological foundations for future CNC screenings [108,109,110,111]. These approaches could be adapted to CNCs once standardized DFT datasets for the five ideal cone geometries are available.

However, the limited size and heterogeneity of the current CNC DFT literature are major constraints. Existing studies have used different model sizes, cone angles, edge passivation schemes, dopants, adsorbates, charge states, and computational settings. Consequently, the available data are not sufficiently standardized for robust ML training. At present, ML models trained directly on published CNC data would risk overfitting to a narrow structural space, particularly because many studies focus on selected cone geometries, H-passivated clusters, and single-adsorbate configurations at 0 K.

A practical near-term strategy is to generate a standardized CNC descriptor database using consistent DFT settings. Such a dataset should include n = 1–5 cone geometries, multiple model sizes, controlled edge passivation, representative dopants, key adsorption sites, and benchmark adsorbates to be useful. Active learning workflows can identify high-uncertainty configurations for targeted DFT calculations, whereas transfer learning from larger graphene and CNT datasets can improve model generalizability [108,109,110,111]. Until such datasets are assembled and validated, ML-accelerated CNC screening should be considered a promising research agenda rather than an immediately deployable predictive tool.

### 15.6. Outlook

Overall, the future of CNC research depends on moving from idealized geometry concepts to experimentally validated structure–property relationships. Theoretical studies have shown that curvature gradients, apex defects, and site-specific adsorption can produce unusual electronic and chemical behaviors [14,16,17,18,20]. However, practical applications require standardized models, controlled synthesis, realistic environmental simulations, quantitative benchmarking, and direct device-level validation.

Until these challenges are addressed, CNCs should be regarded as promising geometry-controlled model systems and potential motifs for cone-rich carbon architectures rather than fully established device materials.

## 16. Application-Validation Status Summary

Table 13 consolidates the validation roadmap. Direct experimental evidence (DE), indirect experimental evidence (IE), and computational prediction (CP) are assigned to a stated claim and architecture according to Section 5.4. A paper can contribute records in more than one class; a CNC-specific calculation remains CP. The final column specifies an experiment or controlled comparison that would test the unresolved inference.

Direct experimental examples include CNC AFM probes [61], conducting cone–particle/adhesive assemblies [64], hollow nanocone-array deionization electrodes [62] and conical carbon-film emitters [29]. These results validate the named structures and functions. They do not, without further controls, validate bulk reinforcement, a molecular sensor, a drug-release device, or the intrinsic property of a selected ideal single-walled cone.

Indirect experimental support includes SWCNH/PVA mechanics [67], PVA-based aerogel insulation [68], PANI/SWCNH transport [69], responsive gels [70], epoxy/CNH electrodes [71], and oxidized-SWCNH/PVP humidity networks [72]. These are actual experimental findings for their materials. They remain indirect evidence when used to motivate claims about isolated, angle-controlled CNCs.

Computational priorities include matched geometry and termination series, charge and reference-state checks, atomistic-to-composite correspondence, and realistic operating environments. Experimental priorities are reproducible material identity, matched filler and processing controls, and an endpoint appropriate to the proposed function. The roadmap does not require a prediction to be treated as validated before those tests are performed.

## 17. Conclusions

Carbon nanocones are useful systems for investigating the relation between apex topology, geometry, edge chemistry, and electronic or adsorption response. The descriptor register defined here makes the source, structural identity, observable, method, and evidence level explicit. Heterogeneous studies establish model-dependent effects; they do not establish one universal numerical ordering across the five ideal apex angles.

Direct experimental evidence is architecture- and endpoint-specific. CNC AFM probes characterize polymer specimens [61]; field-aligned cone–particle assemblies form conducting pathways in an adhesive [64]; hollow nanocone arrays perform capacitive deionization [62]; and conical carbon films demonstrate field emission [29]. These measured outcomes should be retained at the structural level actually tested.

Indirect experimental evidence from nanohorn–polymer materials supports local mechanical reinforcement, insulation, thermoelectric transport, responsive gels, and conducting-network design [67,68,69,70,71,72]. Aggregation, polymer chemistry, orientation, cure or drying history, filler content, and measurement geometry contribute to those results. They cannot be reduced to an isolated cone’s apex angle or used as proof of universal CNC superiority.

Computational prediction remains the principal evidence for controlled-angle electronic tuning, many adsorption and catalytic proposals, CNC–polyethylene interphase mechanics [65], chitosan-associated adsorption [66], model Li-storage capacities [22], and CNC thermal rectification [128]. The revision therefore excludes untraceable pooled gap, work-function, and site-energy intervals and does not infer experimental metallicity, electrode performance or drug release from them.

The validation sequence is to identify the modeled or measured architecture, establish a traceable and comparable descriptor, and test the claimed function against matched controls. Section 16 provides application-specific priorities. For polymer research, the decisive distinctions are probe metrology, interphase modeling, aggregate dispersion, composite transport or mechanics, and biological delivery endpoints. Keeping these distinctions explicit permits useful design hypotheses while preserving the limits of the available evidence.

## Figures and Tables

**Table 1:** Ideal cone geometry derived from Equation (1). Angles are full apex angles rounded to one decimal place; atom counts are not implied by the topological class [11,12,13,14,15,16].

Pentagons n	Removed Sector	sin(α/2)	Full Apex Angle α
1	60°	5/6	112.9°
2	120°	2/3	83.6°
3	180°	1/2	60.0°
4	240°	1/3	38.9°
5	300°	1/6	19.2°

**Table 2:** Key structural differences between ideal/computational carbon nanocones and experimentally accessible single-walled carbon nanohorns. The descriptors were compiled from representative theoretical, computational, and experimental studies on carbon nanocones, atomically precise molecular carboncones, cone-like carbon products, and SWCNH aggregates [11,12,13,14,15,16,17,18,19,54,55,56,57,58,59,60,61,62,63].

Feature	CNC	SWCNH
Structural form	Isolated conical graphene-derived structure with a defined apex topology [11,12,13,14,15,16]	Cone-tipped tubular or horn-like sp^2^-carbon structure [54,55,56,57]
Shape	Open cone with a defined cone angle [11,12,13,14,15,16]	Horn-like tube with a closed or highly curved tip [54,55,56,57]
Aggregation state	Usually treated as an isolated atomistic model [14,16,17,18]	Spherical aggregates, typically tens of nanometers in diameter [54,55,56,57]
Wall structure	Single graphene layer in ideal models [12,14,16]	Single-walled graphitic structure with variable defect density [54,55,56,57]
Cone angle	Discrete set of five ideal angles in topological models [11,12,13,14,15,16]	Distributed and often not individually resolved experimentally [54,55,56,57]
Availability	Mainly computational or observed as heterogeneous cone-like products; atomically precise molecular carboncones have recently been reported [11,12,13,58,59,63]	Experimentally synthesized and widely studied as aggregate powders [54,55,56,57]
Surface area	Model-dependent in finite or atomistic CNC models; not directly comparable to aggregate powders [14,16,17,18]	Generally high accessible surface area; may be modified by oxidation, opening, or activation treatments [54,55,56,57]
Typical role in literature	Often used as atomistically defined model structures for probing geometry-dependent electronic, adsorption, and curvature-related behavior [14,16,17,18,19]	Experimentally accessible aggregate material for synthesis, functionalization, and application studies [54,55,56,57]
Main limitation	Limited direct experimental validation of isolated, angle-controlled CNC structures [13,58,59,60,61,62,63]	Aggregate properties cannot be assigned to isolated cones without caution [54,55,56,57]

**Table 3:** Experimental milestones in carbon nanocones, carbon nanohorns, and related cone-rich carbon research were selected from representative studies [11,12,56,59,61,62,63].

Year	Study	Method	Key Result
1994	Ge and Sattler [12]	Vapor deposition	First observation of fullerene-like carbon cones/carbon nanocones with discrete cone angles
1997	Krishnan et al. [11]	Arc discharge	HRTEM identification of graphitic cones with cone angles consistent with topological predictions
1999	Iijima et al. [56]	CO_2_ laser ablation	Discovery of single-walled carbon nanohorn aggregates, including dahlia-type morphologies
2009	Naess et al. [59]	Pyrolysis	Structural characterization of multi-walled carbon nanocones and related cone-rich carbon products
2019	Zhu et al. [63]	Bottom-up molecular synthesis/mild solution synthesis	Rational synthesis and crystallographic identification of an atomically precise single-layer carboncone molecular scaffold, C_70_H_20_
2024	Paredes et al. [61]	Experimental AFM-based study	Demonstration of carbon nanocones as AFM tips for polymer characterization
2025	Zhang et al. [62]	CVD/electrode growth	Hollow carbon nanocone arrays on carbon fiber cloth for capacitive deionization

**Table 4:** Required fields in the descriptor register and the decision controlled by each field. The register defines a traceable interpretation procedure; it does not combine unlike properties into a single score.

Record Field	Required Entry	Interpretive Decision
Architecture	Isolated/finite CNC, periodic motif, multilayer cone, array, aggregate or composite	Defines the material to which the claim applies
Structure	n and α where meaningful; size, edge termination, defects, charge, adsorbate/site	Prevents unlike structures being treated as one sample
Observable	Quantity, units, equation/reference, total versus per-adsorbate normalization	Prevents unlike descriptors being pooled
Protocol	Functional/potential, dispersion, boundary conditions, convergence, or measurement conditions	Controls the numerical comparison gate
Source	Primary publication and page/table/figure/abstract locator	Permits claim-level checking
Evidence	DE, IE, or CP relative to the stated claim; hypothesis if untested	Separates calculation, proxy measurement, and matched experiment
Comparison status	Matched within-study pair, contextual only, or unavailable	Missing fields do not become assumed values

**Table 5:** Methodological factors affecting the computational predictions for carbon nanocones. The table summarizes the key sources of variability arising from the exchange–correlation functional choice, electronic gap and optical response treatment, finite-size effects, edge passivation, periodic-image artifacts, adsorption-reference conventions, charge-analysis schemes, dispersion corrections, classical force-field approximations, and machine-learning interatomic potentials. Recommended practices are compiled from representative CNC studies, electronic-structure methods, charge analysis and surface-slab protocols, finite-cluster models, dispersion-corrected adsorption benchmarks, atomistic force fields, and machine-learning potential frameworks [14,16,17,18,19,20,21,22,23,24,28,33,49,50,51,52,74,75,77,78,79,80,81,82,83,84,85,86,87,88,89,90,91,92,93,94,95,96,102,103,104,105,106,107,108,109,110,111,112].

**Method Factor**	**Main Issue**	**Expected Impact**	**Recommended Practice**
Exchange–correlation functional	LDA may overestimate binding, whereas semilocal GGA may underestimate weak dispersion-dominated interactions	Adsorption energies, equilibrium distances, and relative stability may vary significantly	Use dispersion-corrected semilocal DFT or benchmark against higher-level methods for physisorption [78,81,82,88,89,90,91,92,93]
Functional choice for electronic gaps	Different functionals and basis sets can yield different HOMO–LUMO gaps, band gaps, and optical transitions	Electronic gaps and optical responses can differ from PBE/LDA or localized-basis values	Report the functional and basis set clearly; avoid comparing gaps from different methods without qualification [14,16,17,18,28,49,50,51,52,74,75,78,102,103,104,105]
Model size	Small clusters, especially very small hydrogen-terminated cones, may be dominated by edge and quantum-confinement effects	Artificially large gaps or edge-localized states may mask genuine curvature effects	Perform size-convergence tests and use larger clusters when possible [14,16,17,18,19,50,51,52,74,75,102,103,104,105]
Edge passivation	Bare edges introduce dangling bonds and possible spin-polarized states	HOMO–LUMO gaps, adsorption energies, and charge-transfer values may be distorted	Use H-passivation for intrinsic apex studies; consider O-functionalized or experimentally realistic edges when appropriate [14,16,17,18,50,51,52,74,75,102,103,104,105]
Periodic image effects	Periodic cone-slab models may suffer from artificial dipoles and image interactions	Work functions, adsorption energies, and charge redistribution may be affected	Use sufficient vacuum spacing, dipole correction, and supercell-convergence tests [77,83,84,85,86,87]
Adsorption reference state	Neutral atom, ion, molecule, reservoir, or bulk reference choices differ across studies	Adsorption energies and voltage-related interpretations may not be directly comparable	Define reference states explicitly and report the adsorption-energy convention [20,21,22,23,24,33,112]
Charge-analysis scheme	Bader, Hirshfeld, Mulliken, and charge-density-difference analyses can yield different values	Charge-transfer magnitudes may differ even for the same system	Use one charge-partitioning method consistently and compare qualitative trends rather than absolute charges alone [94,95,96]
Dispersion treatment	Weak gas and molecular adsorption on sp^2^-carbon surfaces are often dominated by van der Waals interactions; plain GGA may underbind, whereas LDA may overbind at unrealistic distances	Adsorption energies, equilibrium distances, selectivity trends, gas uptake, and adsorption mechanisms may be underestimated or inconsistently described	Use vdW-corrected DFT methods such as DFT-D3/D4, MBD, nonlocal vdW-DF, or rVV10-type functionals, and benchmark weak physisorption trends where possible [88,89,90,91,92,93]
Atomistic force field/empirical potential	Classical potentials can capture large-scale structure but do not directly describe electronic structure, charge transfer, work function, or adsorption energetics	Useful for morphology, stability, and large-scale structural screening, but electronic descriptors require validation	Use empirical or reactive potentials for large systems and validate key energetic and electronic trends with DFT [106,107]
Machine-learning interatomic potential	ML potentials can approach DFT-like structural accuracy within the training domain but may extrapolate poorly for unusual cone angles, apex defects, charged adsorbates, or reactive events	Enables large-scale and long-time simulations, but unreliable extrapolation can distort stability, diffusion, and adsorption trends	Train on diverse DFT data covering cone angles, defects, edges, adsorbates, charge states, and relevant configurations; report training/validation errors and validate key predictions against DFT [108,109,110,111]

**Table 6:** Source-resolved electronic-structure evidence. All rows are computational predictions for the stated structural families. No pooled angle-to-gap or angle-to-work-function values are inferred.

Source	Structural Scope and Method	Reported Finding	Comparison Limit
Charlier and Rignanese [14]	CNC tips; tight-binding and ab initio calculations	Apex resonances depend on pentagon count and relative placement	No extracted common five-angle gap/work-function grid
Compernolle et al. [50]	Small triangle-, square-, and pentagon-apex clusters	Frontier orbitals and gaps depend on apex family and size	Different apex families and cluster sizes
Mattoso et al. [51]	Pentagonal-tip/tip-removed graphannulenes; DFT/B3LYP, neutral/ionic, and Li/F cases	Shell character and size can give oscillatory electronic/adsorption responses	Not a monotonic n = 1–5 angle benchmark
Apriliyanto et al. [52]	Graphannulene topology; Hückel and representative CASSCF calculations	Topological indices organize electronic ground-state character	No common-protocol numerical angle grid
Qu et al. [17]	Defined CNC electronic/field-emission models	Geometry-dependent electronic and emission predictions	A single study is not evidence for the withdrawn five-angle work-function values

**Table 7:** Carbon-nanocone regions and their expected functional roles in adsorption, sensing, ion storage, field emission, and catalytic applications were compiled from representative electronic structure, adsorption, sensing, ion storage, and catalytic studies [17,18,20,21,22,23,24].

Region	Structural Feature	Expected Role
Apex	High curvature and topological defects	Strong adsorption, field enhancement, localized electronic states, and catalytic anchoring [17,18,20,21,22]
Sidewall	Moderately curved graphitic surface	Ion adsorption, molecular interaction, charge transport, and sensing-related adsorption in modified systems [17,18,21,22,23,24]
Hollow site	Multi-carbon coordination site	Alkali-metal adsorption and diffusion [21,22]
Base/edge	Open, passivated, or functionalized boundary	Functionalization, edge states, high reactivity, and modified sensing/catalytic response [18,20,22,23,24]

**Table 8:** Adsorbate identity, structural scope and comparison limits in the cited CNC adsorption literature. The table does not reproduce the untraceable pooled adsorption-energy intervals of the earlier version.

Source and Species	Model or Chemical State	Evidence and Admissible Claim	Excluded Inference
Omidvar [22] Li storage	Pristine CNC-PR and Stone–Wales CNC-SW	CP; same-study defect comparison, capacity/barrier predictions	No verified generic apex/edge/sidewall energy intervals
Hsu et al. [21] Na/K storage	Perfect NC and defective NCD	CP; source-specific cone–anode comparison	No pooled Li/Na/K site-energy hierarchy
Al-Khateeb and El-Barbary [114] Atomic H	Finite H-terminated vacancy-containing CNCs; B3LYP/6-31G	CP; single-H adsorption, distinct vacancy/site models	Not molecular-H_2_ physisorption
Al-Khateeb and El-Barbary [113] Multi-H hydrogenation	Hydrogenated CNC models; total energy referenced to n atomic H	CP; source-defined hydrogenation convention	Not a per-H_2_ adsorption energy
Weak neutral molecular adsorption [88,89,90,91,92,93]	Dispersion-sensitive molecular physisorption; method context	Separate chemical regime requiring matching molecular references	No CNC-specific H_2_ range assigned here

**Table 9:** Source-defined alkali-ion-storage evidence. Numerical CNC entries are the original study’s computational predictions, not measured electrode performance. Cross-material capacities and barriers are not treated as a matched ranking.

Source and Architecture	Study-Defined Quantity	Reported Value or Finding	Evidence and Limit
Graphite LiC_6_ [133]	Conventional stoichiometric capacity	372 mAh g^−1^	Reference composition; not universal graphene or CNC capacity
CNC-PR/CNC-SW [22]	Calculated Li capacity	843/893 mAh g^−1^	CP; pristine/Stone–Wales pair, not measured electrodes
CNC-PR/CNC-SW [22]	Calculated Li migration barrier	0.38/0.32 eV	CP; source-defined pathways, not generic apex/sidewall values
CNC-SW [22]	Reported model open-circuit voltage	0.59 V	CP; model/loading assumptions retained
Perfect/defective cone anodes [21]	Na/K-storage model comparison	Qualitative source-specific discussion	CP; no ion-resolved capacities inferred from ambiguous excerpts
Bud-type nanohorn material [25]	Li-storage experiment	Aggregate-electrode result	IE for isolated CNCs; morphology and contacts matter
Template-derived hard carbon [26]	Na/K-cell experiment	Material-specific electrochemical response	Contextual IE; no curvature-only comparison

**Table 10:** Source-specific sensing and molecular-response evidence. Computational electronic descriptors are separated from a measured nanohorn–polymer network response; neither validates a generic isolated-CNC sensor.

Source and Material	Target and Observable	Evidence	Limit
Kumar et al. [24] Pristine/modified CNC models	ClCN adsorption; calculated electronic-gap changes	CP, electronic-structure calculation	No measured conductance, recovery, or selectivity
Rajabi et al. [23] CNC model	Lomustine binding and electronic descriptors	CP, proposed carrier/sensor context	No experimentally calibrated detection limit
Serban et al. [72] Oxidized-SWCNH/PVP films	Humidity-dependent network resistance and percolation	IE relative to an isolated-CNC sensor claim	Polymer swelling, electrode spacing, and aggregate contacts contribute

**Table 11:** Curvature- and topology-related catalytic applications of carbon nanocones. The table distinguishes direct CNC-specific DFT predictions from indirect evidence based on pentagon-rich, curvature-rich, warped, and nanostructured carbon catalysts [20,33,34,35,36,37,38,149].

Catalytic Process	Carbon/Catalyst System	Main Descriptor or Mechanism	Evidence Level	Ref.
Oxygen reduction reaction, ORR	Warped/disordered graphene-like carbon	Curvature-driven catalytic activity using L/La as a mesoscale descriptor	Experimental/theoretical evidence on disordered curved carbons; indirect mechanistic support for CNCs	[37]
CO_2_ hydrogenation to formic acid	N-doped, Pt-decorated carbon nanocones	N doping and Pt decoration modify CO_2_ activation and the hydrogenation pathway	CP; CNC-specific DFT prediction	[33]
Sulfur-redox chemistry	Curvature-rich N-doped graphene for Li–S batteries	Curvature-induced asymmetric polysulfide redox catalysis	Experimental/theoretical evidence on curvature-rich N-doped graphene; indirect mechanistic support for CNCs	[35]
Water purification	Curved single-atom catalysts	Curved-interface-induced Jahn–Teller effect improves catalytic activity	Experimental/theoretical evidence on curved single-atom catalyst systems; indirect mechanistic support for CNCs	[36]
CH_3_SH photooxidation	Curvature-induced asymmetric Cu–N_2_ structure	Curvature tunes electronic topology and maximizes Cu utilization	Experimental/theoretical evidence on curvature-induced Cu–N_2_ sites; indirect mechanistic support for CNCs	[38]
Single-atom catalysis screening	Carbon-nanocone-supported single-atom sites	High coordination diversity enables screening of optimal catalytic activity	CP; CNC-supported single-atom DFT prediction	[20]
Pentagon-defect catalysis	Pentagon-defect carbon nano-architectures	Pentagon defects act as catalytic sites, especially for ORR-related activity	Review/conceptual and experimental support for pentagon-rich carbons; indirect mechanistic support for CNCs	[34]
Energy electrocatalysis	Curved carbon surfaces and nanostructured carbon catalysts	Surface curvature as an activity descriptor in electrocatalysis	Review/conceptual framework; indirect mechanistic support for CNCs	[149]

**Table 12:** Contextual benchmarks with explicit provenance and limits. The graphene and SWCNT thermal values are published specimen-specific estimates, not transferable CNC constants. No universal work-function, gap or material-performance ranking is inferred.

Source and Specimen	Observable and Conditions	Reported Benchmark	Limit for CNC Comparison
Balandin et al. [129] Suspended single-layer graphene	Room-temperature Raman extraction of thermal conductivity	(4.84 ± 0.44) × 10^3^ to (5.30 ± 0.48) × 10^3^ W m^−1^ K^−1^	Specimen/method-specific; not a CNC or composite value
Pop et al. [130] Suspended metallic SWCNT	Room-temperature conductivity; 2.6 μm length, 1.7 nm diameter	Approximately 3500 W m^−1^ K^−1^	One defined tube; not aggregate effective conductivity
Yang et al. [128] CNC thermal-transport model	Molecular-dynamics rectification over 200–400 K	Computational directional-transport prediction	No experimental CNC diode or transferable conductivity established
Taheri and Fakhrabadi [65] CNC/polyethylene model	MD-derived interphase and finite-element elasticity	Geometry/interface-dependent model response	CP; constitutive and morphology assumptions matter
Knaapila et al. [64] Cone–particle adhesive	Field-aligned conducting assemblies after cure	Measured directional conductivity	DE for that particle network; not intrinsic isolated-cone transport
Nanohorn/PVA and PVA aerogel [67,68]	Indentation mechanics and porous-composite insulation	Experimentally measured, different endpoints	IE for ideal CNCs; polymer, pores, and co-additives contribute

**Table 13:** Application-specific evidence and validation priorities. DE applies to the named, characterized CNC-containing architecture; IE denotes a related material or endpoint that does not validate the same isolated-CNC claim; CP denotes a calculation. A blank experimental claim is not filled by a favorable computational descriptor.

Application	Direct Experimental Evidence DE	Indirect Experimental Evidence IE	Computational Prediction CP	Next Discriminating Test
Polymer metrology	CNC AFM probes [61]	Other nanocarbon probes as context	Mechanical rationale	Probe wear, calibration, and matched-tip controls
Polymer conduction	Cone–particle adhesive assemblies [64]	PANI/epoxy/PVP nanohorn networks [69,71,72]	CNC/PE interphase mechanics [65] concerns a different endpoint	Match loading, dispersion, orientation, and cure; separate contact from intrinsic effects
Composite mechanics and thermal response	No matched isolated-angle CNC dataset identified	PVA/SWNH indentation; PVA aerogel insulation [67,68]	CNC/PE elasticity; CNC thermal rectification [65,128]	Architecture-resolved mechanical/thermal measurements with matched controls
Ion storage	No isolated-CNC battery validation identified	Bud-type nanohorn and hard-carbon electrodes [25,26]	CNC-PR/CNC-SW Li; Na/K cone models [21,22]	Loading/voltage/diffusion consistency and reproducible cycling
Capacitive deionization	Hollow CNC arrays on cloth [62]	Related porous electrodes	Do not infer an atomistic mechanism from a device measurement	Separate area, contact, and cone-geometry effects
Field emission	Conical carbon films [29]	Other carbon emitters [32]	Defined CNC electronic models [17]	Matched tip radius, spacing, and surface work-function tests
Sensing	No isolated-angle CNC gas sensor identified	Oxidized-SWCNH/PVP humidity device [72]	Target-specific CNC adsorption [23,24]	Concentration, humidity, selectivity, recovery, and drift controls
Catalysis	No matched isolated-CNC catalyst test identified	Curved/pentagon-rich supports [34,35,36,37,38]	CNC-supported sites [20,33]	Reaction-specific rates and stability with matched active-site controls
Molecular delivery and optical response	No isolated-CNC release/optical device validated here	Nanocarbon responsive gels [70]	Chitosan-CNC drug adsorption; optical models [28,49,66,150]	Release and biological tests, or measured optical response appropriate to the claim

## Data Availability

No new primary experimental or atomistic-simulation dataset was generated for this review. Retained numerical claims are identified by primary source and locator in Supplementary Table S1. The supporting literature remains available from the cited publications. The package includes the original retained workflow and radar images, revised conceptual illustrations, plotting code for Figure 4 and Figure 6, and input/provenance records. Conceptual atomic networks and curves are not supplied as simulated data.

## Supplementary Materials

The following supporting information can be downloaded at: https://www.mdpi.com/article/10.3390/polym18212644/s1: Table S1, Retained numerical source records; Table S2, Polymer literature by material and evidence; Table S3, Schematic inputs retained from the earlier radar; Figure S1, Schematic-only radar retained from the earlier draft, using Table S3. The supplementary source records refer to [22,64,65,67,68,69,70,71,72,113,114,129,130,133]. Plotting code for Figure 4 and Figure 6, geometric input data, and figure-preparation records accompany the Supporting Files.

## References

[1] Dresselhaus M.S., Dresselhaus G., Avouris P. (2001). Carbon Nanotubes: Synthesis, Structure, Properties, and Applications.

[2] Novoselov K.S., Geim A.K., Morozov S.V., Jiang D., Zhang Y., Dubonos S.V., Grigorieva I.V., Firsov A.A. (2004). Electric field effect in atomically thin carbon films. Science.

[3] Kroto H.W., Heath J.R., O’Brien S.C., Curl R.F., Smalley R.E. (1985). C60: Buckminsterfullerene. Nature.

[4] Saito R., Dresselhaus G., Dresselhaus M.S. (1998). Physical Properties of Carbon Nanotubes.

[5] Castro Neto A.H., Guinea F., Peres N.M.R., Novoselov K.S., Geim A.K. (2009). The electronic properties of graphene. Rev. Mod. Phys..

[6] Lof R.W., van Veenendaal M.A., Koopmans B., Jonkman H.T., Sawatzky G.A. (1992). Band gap, excitons, and Coulomb interaction in solid C60. Phys. Rev. Lett..

[7] Iijima S. (1991). Helical microtubules of graphitic carbon. Nature.

[8] Geim A.K., Novoselov K.S. (2007). The rise of graphene. Nat. Mater..

[9] Robertson J. (1986). Amorphous carbon. Adv. Phys..

[10] Dinadayalane T.C., Leszczynski J. (2010). Remarkable diversity of carbon-carbon bonds: Structures and properties of fullerenes, carbon nanotubes, and graphene. Struct. Chem..

[11] Krishnan A., Dujardin E., Treacy M.M.J., Hugdahl J., Lynum S., Ebbesen T.W. (1997). Graphitic cones and the nucleation of curved carbon surfaces. Nature.

[12] Ge M., Sattler K. (1994). Observation of fullerene cones. Chem. Phys. Lett..

[13] Zhang Q., Xie X.-M., Wei S.-Y., Zhu Z.-Z., Zheng L.-S., Xie S.-Y. (2021). The synthesis of conical carbon. Small Methods.

[14] Charlier J.-C., Rignanese G.-M. (2001). Electronic structure of carbon nanocones. Phys. Rev. Lett..

[15] Terrones M., Terrones H., Hernández E., Grobert N., Charlier J.-C., Ajayan P.M. (2000). New metallic allotropes of planar and tubular carbon. Phys. Rev. Lett..

[16] Lammert P.E., Crespi V.H. (2004). Graphene cones: Classification by fictitious flux and electronic properties. Phys. Rev. B.

[17] Qu C.Q., Qiao L., Wang C., Yu S.S., Zheng W.T., Jiang Q. (2009). Electronic and field emission properties of carbon nanocones: A density functional theory investigation. IEEE Trans. Nanotechnol..

[18] Muñoz-Navia M., Dorantes-Dávila J., Terrones M., Terrones H. (2005). Ground-state electronic structure of nanoscale carbon cones. Phys. Rev. B.

[19] Cox B.J., Hill J.M. (2018). Carbon nanocones with curvature effects close to the vertex. Nanomaterials.

[20] Deng L., Li X., Zhang G., Luo Q., Yang L., Jiang J. (2022). Utilizing high coordination diversity in carbon nanocone supported catalytic single-atom sites for screening of optimal activity. J. Phys. Chem. Lett..

[21] Hsu C.-Y., Ali A.B.M., Jain V., Mohamed A.M.A., Rao D.P., Shomurotova S., Najm M.H., Madhi Z.S., Alkahtani H.M. (2026). Carbon cone-shaped anodes for sodium and potassium-ion batteries: A theoretical study. J. Energy Storage.

[22] Omidvar A. (2020). Defective carbon nanocone as an anode material for lithium-ion batteries. ACS Appl. Energy Mater..

[23] Rajabi N., Pourhakkak P., Jalali Sarvestani M.R. (2025). Exploring the potential of carbon nanocone as nanocarrier and sensor for targeted drug delivery and the detection of lomustine using DFT method. S. Afr. J. Chem. Eng..

[24] Kumar A., Sayyed M.I., Sabugaa M.M., Al-Bahrani M., Sharma S., Saadh M.J. (2023). A DFT study on effective detection of ClCN gas by functionalized, decorated, and doped nanocone strategies. RSC Adv..

[25] Babu D.J., Herdt T., Okeil S., Bruns M., Staudt R., Schneider J.J. (2016). Bud type carbon nanohorns: Materials for high pressure CO2 capture and Li-ion storage. J. Mater. Chem. A.

[26] Igarashi D., Tanaka Y., Kubota K., Tatara R., Maejima H., Hosaka T., Komaba S. (2023). New template synthesis of anomalously large capacity hard carbon for Na- and K-ion batteries. Adv. Energy Mater..

[27] Ghasemi Gol A., Akbari J., Khalaj M., Mousavi-Safavi S.M., Esfahani S., Farahani N. (2023). DFT investigation of a Zn-doped carbon nanocone for the drug delivery of methylated aspirins. Comput. Theor. Chem..

[28] Aloumko’ B., Foadin C.S.T., Ousmanou M.B., Nya F.T., Ejuh G.W. (2023). Enhancing the reactivity, electronic, linear and non-linear optical properties of C_45_H_15_-like carbon nanocone doped with titanium dioxide, boron, and nitrogen: DFT and TD-DFT study. Phys. Scr..

[29] Lu X., Yang Q., Xiao C., Hirose A. (2006). Field electron emission of carbon-based nanocone films. Appl. Phys. A.

[30] Fowler R.H., Nordheim L. (1928). Electron emission in intense electric fields. Proc. R. Soc. Lond. A.

[31] Forbes R.G. (2012). Screened field enhancement factor for a tall closely spaced array of identical conducting posts and implications for Fowler-Nordheim-type equations. J. Appl. Phys..

[32] Bonard J.-M., Salvetat J.-P., Stöckli T., de Heer W.A., Forró L., Châtelain A. (1998). Field emission from single-wall carbon nanotube films. Appl. Phys. Lett..

[33] Injongkol Y., Pimbaotham P., Khemthong P., Wittayakun J., Jungsuttiwong S., Yodsin N. (2025). The impact of nitrogen doping on Pt cluster-decorated carbon nanocones for CO_2_ hydrogenation to formic acid: A DFT study. Fuel.

[34] Chen G., Koide T., Nakamura J., Ariga K. (2025). Nanoarchitectonics for pentagon defects in carbon: Properties and catalytic role in oxygen reduction reaction. Small Methods.

[35] Wang L., Wu C., Gao Y., Du H., Sharma S.K., Marshall A.T., Liu Y., Zhao Y., Dou S., Huang L. (2026). How the curvature-rich structure of N-doped graphene enables asymmetric polysulfide redox catalysis for highly promising Li-S batteries. eScience.

[36] Zhu K., Wang L., Hu Z., Liang X., Zhuang Z., Li X., Yang J., Qiu R., Wang D., Yan K. (2025). Curved interface-induced Jahn-Teller effect in single-atom catalysts for water purification. Nat. Commun..

[37] Kobayashi R., Ishii T., Maie T., Kubota Y., Ozaki J. (2025). Curvature-driven catalytic activity in disordered carbons: L/La as a mesoscale descriptor for ORR catalysis in warped graphene layer structures. Carbon.

[38] Zhong T., Yao Z., Li T., Long X., Zhao H., Tian S., Shu D., He C. (2025). Optimizing electronic topology of curvature-induced Cu-N_2_ asymmetric structure for maximized Cu utilization in CH_3_SH photooxidation. JACS Au.

[39] Avouris P., Freitag M., Perebeinos V. (2008). Carbon-nanotube photonics and optoelectronics. Nat. Photonics.

[40] Li Z.Q., Henriksen E.A., Jiang Z., Hao Z., Martin M.C., Kim P., Stormer H.L., Basov D.N. (2008). Dirac charge dynamics in graphene by infrared spectroscopy. Nat. Phys..

[41] Wang F., Zhang Y., Tian C., Girit C., Zettl A., Crommie M., Shen Y.R. (2008). Gate-variable optical transitions in graphene. Science.

[42] Yang L., Deslippe J., Park C.-H., Cohen M.L., Louie S.G. (2009). Excitonic effects on the optical response of graphene and bilayer graphene. Phys. Rev. Lett..

[43] Minaie B., Ketabi S.A., De Sousa J.M. (2025). Structural, electronic and optical properties of penta-graphene nanotubes (PGNTs): GW-BSE approach. Appl. Phys. A.

[44] Gopalakrishnan C., Kamalakannan S., Ham H.C., Al-Mogren M.M., Hochlaf M., Prakash M. (2025). Controlling electronic and optoelectronic behavior of graphene ionic liquids based metal salt amalgamation: A DFT perspective. SSRN.

[45] Blase X., Benedict L.X., Shirley E.L., Louie S.G. (1994). Hybridization effects and metallicity in small radius carbon nanotubes. Phys. Rev. Lett..

[46] Ajiki H., Ando T. (1993). Electronic states of carbon nanotubes. J. Phys. Soc. Jpn..

[47] Spataru C.D., Ismail-Beigi S., Benedict L.X., Louie S.G. (2004). Excitonic effects and optical spectra of single-walled carbon nanotubes. Phys. Rev. Lett..

[48] Marinopoulos A.G., Reining L., Olevano V., Rubio A., Pichler T., Liu X., Knupfer M., Fink J. (2002). Anisotropy and interplane interactions in the dielectric response of graphite. Phys. Rev. Lett..

[49] Zhang J., Zhang F.-Y., Su Z.-M., Xu H.-L. (2022). Pyramid-like Au_2_-CNC under an external electric field: Charge transfer, uv-vis absorption spectra, and nonlinear optical property. J. Phys. Chem. C.

[50] Compernolle S., Kiran B., Chibotaru L.F., Nguyen M.T., Ceulemans A. (2004). Ab initio study of small graphitic cones with triangle, square, and pentagon apex. J. Chem. Phys..

[51] Mattoso S.H., Brumas V., Evangelisti S., Fronzoni G., Leininger T., Stener M. (2023). Electronic structure of pentagonal carbon nanocones: An ab initio study. J. Phys. Chem. A.

[52] Apriliyanto Y.B., Battaglia S., Evangelisti S., Faginas-Lago N., Leininger T., Lombardi A. (2021). Toward a generalized Hückel rule: The electronic structure of carbon nanocones. J. Phys. Chem. A.

[53] Jiménez-Soto J.M., Cárdenas Aranzana M.S., Meyers R.A. (2012). Conical Carbon Nanoparticles in Analytical Chemistry. Encyclopedia of Analytical Chemistry.

[54] Yudasaka M., Iijima S., Crespi V.H., Jorio A., Dresselhaus G., Dresselhaus M.S. (2008). Single-wall carbon nanohorns and nanocones. Carbon Nanotubes.

[55] Karousis N., Suarez-Martinez I., Ewels C.P., Tagmatarchis N. (2016). Structure, Properties, Functionalization, and Applications of Carbon Nanohorns. Chem. Rev..

[56] Iijima S., Yudasaka M., Yamada R., Bandow S., Suenaga K., Kokai F., Takahashi K. (1999). Nano-aggregates of single-walled graphitic carbon nano-horns. Chem. Phys. Lett..

[57] Zhu S., Xu G. (2010). Single-walled carbon nanohorns and their applications. Nanoscale.

[58] Lin C.-T., Lee C.-Y., Chiu H.-T., Chin T.-S. (2007). Graphene structure in carbon nanocones and nanodiscs. Langmuir.

[59] Naess S.N., Elgsaeter A., Helgesen G., Knudsen K.D. (2009). Carbon nanocones: Wall structure and morphology. Sci. Technol. Adv. Mater..

[60] Dimovski S., Libera J.A., Gogotsi Y. (2001). A Novel Class of Carbon Nanocones. MRS Proc..

[61] Paredes G., Villeneuve-Faure C., Monthioux M. (2024). *Carbon* nanocone mechanical behavior and their use as AFM tips for the characterization of polymers in Peak Force mode. Carbon.

[62] Zhang M., Lu X., Kong W., Jin X., Tan K., Wang Y., Feng Y. (2025). Hollow carbon nanocone arrays on carbon fiber cloth as a free-standing electrode for high-performance capacitive deionization. Sep. Purif. Technol..

[63] Zhu Z.-Z., Chen Z.-C., Yao Y.-R., Cui C.-H., Li S.-H., Zhao X.-J., Zhang Q., Tian H.-R., Xu P.-Y., Xie F.-F. (2019). Rational synthesis of an atomically precise carboncone under mild conditions. Sci. Adv..

[64] Knaapila M., Rømoen O.T., Svåsand E., Pinheiro J.P., Martinsen Ø.G., Buchanan M., Skjeltorp A.T., Helgesen G. (2011). Conductivity Enhancement in Carbon Nanocone Adhesive by Electric Field Induced Formation of Aligned Assemblies. ACS Appl. Mater. Interfaces.

[65] Taheri S.S., Fakhrabadi M.M.S. (2022). Interphase effects on elastic properties of polymer nanocomposites reinforced by carbon nanocones. Comput. Mater. Sci..

[66] Hsu C.-Y., Kareem R.A., Ruziyev O., Jafarova A.M., Mirzaei M. (2025). DFT assessments on a chitosan-modified nanocone scaffold for the adsorption of ciclopirox antifungal drug to provide insights into the engineering a potential nano-based drug delivery platform. Int. J. Nano Dimens..

[67] Kadambi S.B., Pramoda K., Ramamurty U. (2015). Carbon-Nanohorn-Reinforced Polymer Matrix Composites: Synergetic Benefits in Mechanical Properties. ACS Appl. Mater. Interfaces.

[68] Xu J., Ao X., de la Vega J., Guo F., Xie Z., Liang F., Wang D.-Y., Wu J. (2024). Poly(vinyl alcohol) Composite Aerogel toward Lightweight, Remarkable Flame Retardancy, and Thermal Insulation Properties by Incorporating Carbon Nanohorns and Phytic Acid. ACS Appl. Polym. Mater..

[69] Famengo A., Ferrario A., Boldrini S., Battiston S., Fiameni S., Pagura C., Fabrizio M. (2017). Polyaniline–carbon nanohorn composites as thermoelectric materials. Polym. Int..

[70] Miyako E., Nagata H., Hirano K., Hirotsu T. (2008). Photodynamic Thermoresponsive Nanocarbon–Polymer Gel Hybrids. Small.

[71] Montes R., Baeza M., Muñoz J. (2020). 0D polymer nanocomposite carbon-paste electrodes using carbon nanohorns: Percolating networks, electrochemical achievements and filler comparison. Compos. Sci. Technol..

[72] Serban B.-C., Cobianu C., Dumbravescu N., Buiu O., Bumbac M., Nicolescu C.M., Cobianu C., Brezeanu M., Pachiu C., Serbanescu M. (2021). Electrical Percolation Threshold and Size Effects in Polyvinylpyrrolidone-Oxidized Single-Wall Carbon Nanohorn Nanocomposite: The Impact for Relative Humidity Resistive Sensors Design. Sensors.

[73] Dresselhaus M.S., Dresselhaus G., Eklund P.C. (1996). Science of Fullerenes and Carbon Nanotubes.

[74] Tamura R., Tsukada M. (1994). Disclinations of monolayer graphite and their electronic states. Phys. Rev. B.

[75] El-Barbary A.A., Khurmy S.M., Adawi H. (2025). Band gap engineering of nanocones via mono-hydrogenation. Macromol. Symp..

[76] Murata K., Kaneko K., Steele W.A., Kokai F., Takahashi K., Kasuya D., Yudasaka M., Iijima S. (2001). Molecular potential structures of heat-treated single-wall carbon nanohorn assemblies. J. Phys. Chem. B.

[77] Bengtsson L. (1999). Dipole correction for surface supercell calculations. Phys. Rev. B.

[78] Perdew J.P., Burke K., Ernzerhof M. (1996). Generalized gradient approximation made simple. Phys. Rev. Lett..

[79] Blöchl P.E. (1994). Projector augmented-wave method. Phys. Rev. B.

[80] Kresse G., Joubert D. (1999). From ultrasoft pseudopotentials to the projector augmented-wave method. Phys. Rev. B.

[81] Hohenberg P., Kohn W. (1964). Inhomogeneous electron gas. Phys. Rev..

[82] Kohn W., Sham L.J. (1965). Self-consistent equations including exchange and correlation effects. Phys. Rev..

[83] Kresse G., Furthmüller J. (1996). Efficient iterative schemes for ab initio total-energy calculations using a plane-wave basis set. Phys. Rev. B.

[84] Kresse G., Furthmüller J. (1996). Efficiency of ab-initio total energy calculations for metals and semiconductors using a plane-wave basis set. Comput. Mater. Sci..

[85] Kresse G., Hafner J. (1993). Ab initio molecular dynamics for liquid metals. Phys. Rev. B.

[86] Kresse G., Hafner J. (1994). Ab initio molecular-dynamics simulation of the liquid-metal–amorphous-semiconductor transition in germanium. Phys. Rev. B.

[87] Blöchl P.E., Jepsen O., Andersen O.K. (1994). Improved tetrahedron method for Brillouin-zone integrations. Phys. Rev. B.

[88] Grimme S., Antony J., Ehrlich S., Krieg H. (2010). A consistent and accurate ab initio parametrization of density functional dispersion correction (DFT-D) for the 94 elements H–Pu. J. Chem. Phys..

[89] Brandenburg J.G., Zen A., Alfè D., Michaelides A. (2019). Interaction between water and carbon nanostructures: How good are current density functional approximations?. J. Chem. Phys..

[90] Krishnan S., Vadapoo R., Riley K.E., Velev J.P. (2011). Dispersion-corrected density functional theory comparison of hydrogen adsorption on boron-nitride and carbon nanotubes. Phys. Rev. B.

[91] Chowdhury S., Tang H., Perdew J.P. (2021). van der Waals corrected density functionals for cylindrical surfaces: Ammonia and nitrogen dioxide adsorbed on a single-walled carbon nanotube. Phys. Rev. B.

[92] Ershova O.V., Lillestolen T.C., Bichoutskaia E. (2010). Study of polycyclic aromatic hydrocarbons adsorbed on graphene using density functional theory with empirical dispersion correction. Phys. Chem. Chem. Phys..

[93] Ulbricht H., Zacharia R., Cindir N., Hertel T. (2006). Thermal desorption of gases and solvents from graphite and carbon nanotube surfaces. Carbon.

[94] Henkelman G., Arnaldsson A., Jónsson H. (2006). A fast and robust algorithm for Bader decomposition of charge density. Comput. Mater. Sci..

[95] Bader R.F.W. (1991). A quantum theory of molecular structure and its applications. Chem. Rev..

[96] Tang W., Sanville E., Henkelman G. (2009). A grid-based Bader analysis algorithm without lattice bias. J. Phys. Condens. Matter.

[97] Shiraishi M., Ata M. (2001). Work function of carbon nanotubes. Carbon.

[98] Yu Y.-J., Zhao Y., Ryu S., Brus L.E., Kim K.S., Kim P. (2009). Tuning the graphene work function by electric field effect. Nano Lett..

[99] Shan B., Cho K. (2005). First principles study of work functions of single wall carbon nanotubes. Phys. Rev. Lett..

[100] Su W.S., Leung T.C., Li B., Chan C.T. (2007). Work function of small radius carbon nanotubes and their bundles. Appl. Phys. Lett..

[101] Su W.S., Leung T.C., Li B., Chan C.T. (2007). Work function of single-walled and multiwalled carbon nanotubes: First-principles study. Phys. Rev. B.

[102] Chen J.-L., Su M.-H., Hwang C.-C., Lu J.-M., Tsai C.-C. (2010). Low-Energy electronic states of carbon nanocones in an electric field. Nano-Micro Lett..

[103] Gomes F.A., Bezerra V.B., de Lima J.R.F., Moraes F.J.S. (2019). Electronic properties of single and double napped carbon nanocones. Eur. Phys. J. B.

[104] Brito E., Freitas A., Silva T., Guerra T., Azevedo S. (2015). Double-walled carbon nanocones: Stability and electronic structure. Eur. Phys. J. B.

[105] Ming C., Lin Z.-Z., Zhuang J., Ning X.-J. (2012). Electronic rectification devices from carbon nanocones. Appl. Phys. Lett..

[106] van Duin A.C.T., Dasgupta S., Lorant F., Goddard W.A. (2001). ReaxFF: A reactive force field for hydrocarbons. J. Phys. Chem. A.

[107] Tersoff J. (1989). Modeling solid-state chemistry: Interatomic potentials for multicomponent systems. Phys. Rev. B.

[108] Smith J.S., Isayev O., Roitberg A.E. (2017). ANI-1: An extensible neural network potential with DFT accuracy at force field computational cost. Chem. Sci..

[109] Rowe P., Csányi G., Alfè D., Michaelides A. (2018). Development of a machine learning potential for graphene. Phys. Rev. B.

[110] Vandermause J., Torrisi S.B., Batzner S., Xie Y., Sun L., Kolpak A.M., Kozinsky B. (2020). On-the-fly active learning of interpretable Bayesian force fields for atomistic rare events. npj Comput. Mater..

[111] Batzner S., Musaelian A., Sun L., Geiger M., Mailoa J.P., Kornbluth M., Molinari N., Smidt T.E., Kozinsky B. (2022). E(3)-equivariant graph neural networks for data-efficient and accurate interatomic potentials. Nat. Commun..

[112] Neugebauer J., Scheffler M. (1992). Adsorbate-substrate and adsorbate-adsorbate interactions of Na and K adlayers on Al(111). Phys. Rev. B.

[113] Al-Khateeb M.A., El-Barbary A.A. (2026). Irradiation-enhanced hydrogen storage in vacancy-engineered carbon and boron nitride nanocones: A coupled DFT and PHITS analysis. J. Radiat. Res. Appl. Sci..

[114] Al-Khateeb M.A., El-Barbary A.A. (2026). Multiscale investigation of hydrogen adsorption in vacancy-engineered CNCs: A combined DFT and PHITS Monte Carlo study. Open J. Compos. Mater..

[115] Charlier J.-C., Blase X., Roche S. (2007). Electronic and transport properties of nanotubes. Rev. Mod. Phys..

[116] Dresselhaus M.S., Dresselhaus G., Saito R. (1995). Physics of carbon nanotubes. Carbon.

[117] Lee C., Wei X., Kysar J.W., Hone J. (2008). Measurement of the elastic properties and intrinsic strength of monolayer graphene. Science.

[118] Treacy M.M.J., Ebbesen T.W., Gibson J.M. (1996). Exceptionally high Young’s modulus observed for individual carbon nanotubes. Nature.

[119] Falvo M.R., Clary G.J., Taylor R.M., Chi V., Brooks F.P., Washburn S., Superfine R. (1997). Bending and buckling of carbon nanotubes under large strain. Nature.

[120] Yeh N.-C., Hsu C.-C., Teague M.L., Wang J.-Q., Boyd D.A., Chen C.-C. (2016). Nanoscale strain engineering of graphene and graphene-based devices. Acta Mech. Sin..

[121] Wakker G.M.M., Tiwari R.P., Blaauboer M. (2011). Localization and circulating currents in curved graphene devices. Phys. Rev. B.

[122] Mahmud M.T., Sandler N. (2020). Emergence of strain-induced Moiré patterns and pseudomagnetic field confined states in graphene. Phys. Rev. B.

[123] Tran V.-T., Saint-Martin J., Dollfus P. (2020). Electron transport properties of graphene nanoribbons with Gaussian deformation. Phys. Rev. B.

[124] Ling F., Liao R., Yuan C., Shi X., Li L., Zhou X., Tang X., Jing C., Wang Y., Jiang S. (2023). Geometric, electronic and transport properties of bulged graphene: A theoretical study. J. Chem. Phys..

[125] Pacheco Sanjuan A.A., Wang Z., Imani H.P., Vanević M., Barraza-Lopez S. (2014). Graphene’s morphology and electronic properties from discrete differential geometry. Phys. Rev. B.

[126] Giambastiani D., Colangelo F., Tredicucci A., Roddaro S., Pitanti A. (2022). Electron localization in periodically strained graphene. J. Appl. Phys..

[127] Santos M.C., da Silva E.L., Baptista D.S., Santos T., Molinari M., Manjón F.J., Cui Y., Lin X., Yang T. (2026). Strain-induced curvature in monolayer graphene: Effects on electronic structure, phonon dynamics, and lattice thermal conductivity. arXiv.

[128] Yang N., Zhang G., Li B. (2008). Carbon nanocone: A promising thermal rectifier. Appl. Phys. Lett..

[129] Balandin A.A., Ghosh S., Bao W., Calizo I., Teweldebrhan D., Miao F., Lau C.N. (2008). Superior thermal conductivity of single-layer graphene. Nano Lett..

[130] Pop E., Mann D., Wang Q., Goodson K., Dai H. (2006). Thermal conductance of an individual single-wall carbon nanotube above room temperature. Nano Lett..

[131] Kahn A., Koch N., Gao W. (2003). Electronic structure and electrical properties of interfaces between metals and π-conjugated molecular films. J. Polym. Sci. Part B Polym. Phys..

[132] Braun S., Salaneck W.R., Fahlman M. (2009). Energy-level alignment at organic/metal and organic/organic interfaces. Adv. Mater..

[133] Grosu C., Panosetti C., Merz S., Jakes P., Seidlmayer S., Matera S., Eichel R.-A., Granwehr J., Scheurer C. (2023). Revisiting the storage capacity limit of graphite battery anodes: Spontaneous lithium overintercalation at ambient pressure. PRX Energy.

[134] Giovannetti G., Khomyakov P.A., Brocks G., Karpan V.M., van den Brink J., Kelly P.J. (2008). Doping graphene with metal contacts. Phys. Rev. Lett..

[135] Marinopoulos A.G., Reining L., Rubio A., Olevano V. (2004). Ab initio study of the optical absorption and wave-vector-dependent dielectric response of graphite. Phys. Rev. B.

[136] Venghaus H. (1977). Infrared reflectance and dielectric properties of pyrolytic graphite for E || c polarization. Phys. Status Solidi B.

[137] Zeppenfeld K. (1971). Nichtsenkrechte interbandübergänge in graphit durch unelastische elektronenstreuung. Z. Phys..

[138] Marinopoulos A.G., Reining L., Rubio A., Vast N. (2003). Optical and loss spectra of carbon nanotubes: Depolarization effects and intertube interactions. Phys. Rev. Lett..

[139] Mak K.F., Sfeir M.Y., Wu Y., Lui C.H., Misewich J.A., Heinz T.F. (2008). Measurement of the optical conductivity of graphene. Phys. Rev. Lett..

[140] Kataura H., Kumazawa Y., Maniwa Y., Umezu I., Suzuki S., Ohtsuka Y., Achiba Y. (1999). Optical properties of single-wall carbon nanotubes. Synth. Met..

[141] Fink J., Müller-Heinzerling T., Pflüger J., Scheerer B., Dischler B., Koidl P., Bubenzer A., Sah R.E. (1984). Investigation of hydrocarbon-plasma-generated carbon films by electron-energy-loss spectroscopy. Phys. Rev. B.

[142] Eberlein T., Bangert U., Nair R.R., Jones R., Gass M., Bleloch A.L., Novoselov K.S., Geim A., Briddon P.R. (2008). Plasmon spectroscopy of free-standing graphene films. Phys. Rev. B.

[143] Grüneis A., Saito R., Samsonidze Ge.G., Kimura T., Pimenta M.A., Jorio A., Souza Filho A.G., Dresselhaus G., Dresselhaus M.S. (2003). Inhomogeneous optical absorption around the K point in graphite and carbon nanotubes. Phys. Rev. B.

[144] Saito R., Dresselhaus G., Dresselhaus M.S. (2000). Trigonal warping effect of carbon nanotubes. Phys. Rev. B.

[145] Bonaccorso F., Sun Z., Hasan T., Ferrari A.C. (2010). Graphene photonics and optoelectronics. Nat. Photonics.

[146] Garcia de Abajo F.J. (2014). Graphene plasmonics: Challenges and opportunities. ACS Photonics.

[147] Onida G., Reining L., Rubio A. (2002). Electronic excitations: Density-functional versus many-body Green’s-function approaches. Rev. Mod. Phys..

[148] Rohlfing M., Louie S.G. (2000). Electron-hole excitations and optical spectra from first principles. Phys. Rev. B.

[149] Wang Q., Lyu L., Pan H., Wang Z., Lee D., Liu J., Li Z., Lei Y., Kang Y.-M. (2025). From geometry to activity: Understanding surface curvature effects in energy electrocatalysis. ACS Mater. Lett..

[150] Salba, Naseem Z., Ayub A.R., Arshed S.M., Afzal F., Arif S., Raza U., Zubair S., Arif K., Hamid H. (2026). Carbon nanocone oxide-mediated controlled interaction to increase Favipiravir’s bioavailability: An extensive in silico research. Comput. Biol. Chem..

