# Supplementary material for: Carbon Nanocones Geometry-Dependent Descriptors Evidence Levels and Polymer Interfaces
Source: Polymers (Basel). 2026 Oct 25;18(21):2644. doi: 10.3390/polym18212644 (PMC13766146; doi:10.3390/polym18212644)
Supplement: Supplementary file 1 [file polymers-18-02644-s001.zip › polymers-4648996-supplementary.pdf]

Supplementary Materials

Carbon Nanocones Geometry Dependent Descriptors Evidence Levels and Polymer Interfaces

Can Doğan Vurdu and Fatma Kandemirli

Reference numbers correspond to the revised main manuscript. No new primary experimental or atomistic-simulation data are introduced here. Source records, geometry calculations and schematic-only inputs serve different purposes and are kept separate.

Table S1 Retained numerical source records

Primary source and locator	Quantity and reported result	Model or measurement conditions	Interpretation limit
Omidvar [22]<br>Abstract, p. 11463<br>10.1021/acsaem.0c02457	Li capacity: CNC-PR 843; CNC-SW 893 mAh g^{-1}	Pristine versus Stone–Wales-defected CNC model pair; DFT prediction	Calculated capacity; no experimental CNC electrode validation. Method/reference details not reconstructed from the abstract.
Omidvar [22]<br>Abstract, p. 11463	Li migration barriers: 0.38 / 0.32 eV; CNC-SW OCV 0.59 V	Source-defined CNC-PR/CNC-SW pathways and loading model	Not generic apex/sidewall barrier or universal voltage.
Grosu et al. [133]<br>Introduction<br>10.1103/PRXEnergy.2.013003	Conventional LiC_6 capacity: 372 mAh g^{-1}	Graphite stoichiometry reference	Not a measured or theoretical capacity assigned to all graphene or CNC structures.
Balandin et al. [129]<br>Abstract, p. 902<br>10.1021/nl0731872	Thermal conductivity estimates: $(4.84 \pm 0.44) \times 10^3$ to $(5.30 \pm 0.48) \times 10^3$ W m^{-1} K^{-1}	Suspended single-layer graphene; room-temperature Raman-based extraction	Original reported estimates with their uncertainties; no pooling with CNCs or composite conductivity.
Pop et al. [130]<br>Abstract, p. 96<br>10.1021/nl052145f	Approximately 3500 W m^{-1} K^{-1} at room temperature	Suspended metallic SWCNT; length 2.6 μm, diameter 1.7 nm; Joule-heating I–V analysis	Specimen- and model-dependent extraction, not an intrinsic value for CNC aggregates.

The earlier five-angle gap/work-function table and Li site-energy interval table are withdrawn. Their values lacked source-by-value model assignments. The cited atomic-H and multi-H hydrogenation studies [113,114] are not a molecular-H_2 physisorption dataset. Missing quantities are not filled by representative midpoints or illustrative ranges.

Table S2 Polymer literature by material and evidence

Source	Architecture and endpoint	Evidence relative to an isolated CNC claim	Critical limit
Taheri et al. [65] 10.1016/j.commatsci.2021.110910	CNC/polyethylene; MD-derived interphases and FE elasticity	CP; direct CNC model	Interphase constitutive and morphology assumptions; no measured reinforcement.
Knaapila et al. [64] 10.1021/am100990c	Cone-particle adhesive; field-aligned conducting assemblies	DE for the tested cone-particle network	Multilayer/particle assembly and processing; not intrinsic isolated-cone conductivity.
Kadambi et al. [67] 10.1021/acsami.5b02792	PVA/SWNH; nanoindentation modulus and hardness	IE for an isolated CNC	Local indentation response, not tensile strength or angle-controlled CNC reinforcement.
Xu et al. [68] 10.1021/acsapm.4c00729	PVA/phytic-acid/SWCNH aerogel; mechanics and insulation	IE for an isolated CNC	Porosity and co-additive act together; low conductivity is an insulation outcome.
Famengo et al. [69] 10.1002/pi.5366	Doped PANI/SWCNH; electrical, Seebeck and thermal transport	IE for an isolated CNC	Composition, PANI doping and film/pellet geometry matter.
Miyako et al. [70] 10.1002/smll.200800601	SWNH/SWNT-containing agarose and PNIPAM; NIR-driven transitions	IE for an isolated CNC	Responsive-gel functionality, not therapeutic release or mechanical reinforcement.
Montes et al. [71] 10.1016/j.compscitech.2020.108260	Epoxy/CNH carbon-paste electrode; percolating electroactive network	IE for an isolated CNC	Filler/polymer ratio and aggregate contacts control the device response.
Serban et al. [72] 10.3390/s21041435	Oxidized-SWCNH/PVP; humidity resistance and electrode-spacing effects	IE for an isolated CNC	Apparent threshold depends on device geometry and swelling, not only filler identity.

DE and IE are relative to the stated claim: a nanohorn–polymer measurement is direct evidence for that nanohorn composite and indirect when used to support a proposed isolated-CNC outcome. The main text identifies the experimental architecture instead of treating all cone-like carbons as one material.

Table S3 Schematic inputs retained from the earlier radar

These supplied values are an uncalibrated schematic. No reproducible empirical criteria, weights, uncertainty model or validation supports the 1–10 scale. They are not used by the descriptor register, DE/IE/CP classification, quantitative comparisons or conclusions. No property or maturity ranking should be inferred from them.

Schematic label	Li-binding axis	Low-work-function axis	Gap-tunability axis	Experiment axis	Synthesis axis
CNC n = 5	10	9	8	2	2
CNC n = 3	8	7	7	2	2
Graphene	4	4	5	10	9
SWCNT	5	5	6	8	8
C60	6	3	7	7	7

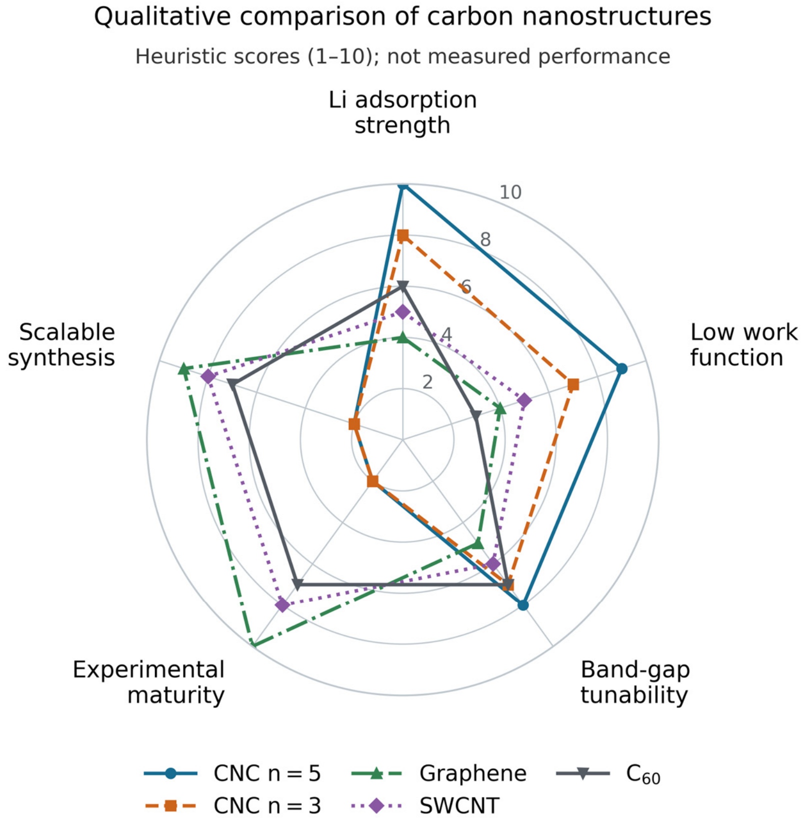

Figure S1. Schematic-only radar retained from the earlier draft, using Table S3. Axes and values are illustrative choices, not calibrated measurements, a literature meta-analysis or a reproducible performance score. The chart is excluded from the main manuscript's analysis and conclusions.
